# Two centuries from species discovery to diagnostic characters: molecular and morphological evidence for narrower species limits in the widespread SW Australian *Anarthria gracilis* complex (Restionaceae s.l./Anarthriaceae, Poales)

**DOI:** 10.7717/peerj.10935

**Published:** 2021-03-08

**Authors:** Constantin I. Fomichev, Terry D. Macfarlane, Carmen M. Valiejo-Roman, Tahir H. Samigullin, Galina V. Degtjareva, Barbara G. Briggs, Dmitry D. Sokoloff

**Affiliations:** 1 Department of Higher Plants, Biological Faculty, M.V. Lomonosov Moscow State University, Moscow, Russia; 2 Western Australian Herbarium, Biodiversity and Conservation Science, Department of Biodiversity, Conservation and Attractions, Kensington, WA, Australia; 3 Department of Evolutionary Biochemistry, A.N. Belozersky Institute of Physico-Chemical Biology, M.V. Lomonosov Moscow State University, Moscow, Russia; 4 Botanical Garden, Biological Faculty, M.V. Lomonosov Moscow State University, Moscow, Russia; 5 National Herbarium of New South Wales, Royal Botanic Gardens Trust, Sydney, NSW, Australia

**Keywords:** Restionaceae, Poales, Anarthria, Western Australia, Phylogeny, Leaf morphology, Leaf ligule, Diagnostic characters, Micromorphology, South-western Australian biodiversity hotspot

## Abstract

**Background:**

The extreme southwest of Australia is a biodiversity hotspot region that has a Mediterranean-type climate and numerous endemic plant and animal species, many of which remain to be properly delimited. We refine species limits in *Anarthria*, a Western Australian endemic genus characterised by the occurrence of the greatest number of plesiomorphic character states in the restiid clade of Poales. In contrast to many other groups of wind-pollinated Australian Poales, *Anarthria* was traditionally viewed as having well-established species limits. All six currently recognised species, which are conspicuous members of some Western Australian plant communities, were described in the first half of the 19th century. They are traditionally distinguished from each other mainly using quantitative characters.

**Methods:**

We examined extensive existing herbarium specimens and made new collections of *Anarthria* in nature. Scanning electron microscopy and light microscopy were used to study leaf micromorphology. Molecular diversity of *Anarthria* was examined using a plastid (*trn*L-F) and a low-copy nuclear marker (*at*103). This is the first study of species-level molecular diversity in the restiid clade using a nuclear marker.

**Results:**

Material historically classified as *Anarthria gracilis* R.Br. actually belongs to three distinct species, *A. gracilis* s.str., *A. grandiflora* Nees and *A. dioica* (Steud.) C.I.Fomichev, each of which forms a well-supported clade in phylogenetic analyses. Both segregate species were described in the first half of the 19th century but not recognised as such in subsequent taxonomic accounts. *Anarthria dioica* was first collected in 1826, then wrongly interpreted as a species of *Juncus* (Juncaceae) and described as *Juncus dioicus*. We provide a formal transfer of the name to *Anarthria* and for the first time report its clear and qualitative diagnostic characters: an extremely short leaf ligule and distinctive pattern of leaf epidermal micromorphology. A long ligule is present in *A. gracilis* s.str. and *A. grandiflora*. These species differ from each other in leaf lamina morphology and anatomy and have mostly non-overlapping distribution ranges. The narrower definition of species provides a basis for future phylogeographic analyses in *Anarthria*. Our study highlights a need for more extensive use of nuclear DNA markers in Restionaceae. The use of the low copy nuclear marker *at*103 allowed a clade comprising all three ligulate species of *Anarthria* to be recognised. The ligule character is used here for the first time in the taxonomy of *Anarthria* and merits special attention in studies of other restiids. In general, our study uncovered a superficially hidden but, in reality, conspicuous diversity in a common group of wind-pollinated plants in the southwest of Western Australia.

## Introduction

Most species of the restiid clade (Restionaceae sensu APG IV, 2016), which is sister to the graminid clade within the order Poales (Linder & Rudall, 2005; Briggs, Marchant & Perkins, 2010, 2014; Givnish et al., 2010, 2018) occur in the Cape region of South Africa and in Australia, where they are an important part of the vegetation (Linder, Briggs & Johnson, 1998). Though species-rich, the family is well-known as difficult in species identification for botanists not directly specialised in the group. *Anarthria* R.Br., *Lyginia* R.Br. and *Hopkinsia* W.Fitzg. form a clade (Anarthriaceae sensu Council of Heads of Australasian Herbaria, 2006–2021; Briggs, Marchant & Perkins, 2014) that is a sister group to all other restiids and differs from them in dithecal rather than monothecal anthers (Briggs et al., 2000; APG II, 2003; Briggs, Marchant & Perkins, 2010, 2014). The three genera of dithecal restiids comprise in total eleven currently recognised species (Briggs, 2020), all endemic to the southwest of Western Australia. *Anarthria* is the largest genus of the group with six currently recognised species. The species of *Anarthria* grow on oligotrophic sandy or peaty soils of seasonally damp heath, woodlands (sometimes along stream banks) or swamps in regions of Mediterranean climate mainly in the south west of the continent from Esperance to Eneabba (Meney & Pate, 1999; Briggs, Connelly & Krauss, 2020a). The genus *Anarthria* has the most symplesiomorphies in restiids, such as dithecal anthers, trimerous flowers, long ovules and leaves with well-developed laminae (Meney & Pate, 1999; Fomichev et al., 2019).

Except for the distinctive species *A. prolifera* R.Br., the species of *Anarthria* are distinguished from each other mostly by quantitative features such as stem diameter, leaf width, plant height, inflorescence length, flower number per male and female inflorescence and flower length (Meney & Pate, 1999; Briggs, 2020). In contrast to many other groups of wind-pollinated Poales with centres of diversity in Australia, *Anarthria* was traditionally considered as a genus with well-established species limits, though segregation of *A. humilis* Nees from *A. gracilis* R.Br. was accepted in herbarium practice since the 1960s but not in printed literature until the 1980s (Green, 1981; Rye, 1987). Species of the genus are common and conspicuous in some parts of the southwest of Western Australia and can be easily distinguished from each other in the field when more than one species grow together in the same locality. All six currently recognised species of *Anarthria* were described during an early phase of exploration of the Australian flora (Brown, 1810; Nees von Esenbeck, 1841, 1846).

During field work in the southwest of Western Australia, three of us (CIF, TDM and DDS) noticed that some plants fitting the traditional taxonomic circumscription of *A*. *gracilis* R.Br. possessed a ligule about 1 cm long whereas others had a barely visible ligule in the form of extremely narrow joining margins of the leaf sheath. Finding clear and abrupt differences in such a well-defined qualitative character was an unexpected observation, so we explored the taxonomic diversity of *Anarthria* more fully. Cutler & Airy Shaw (1965) and Cutler (1969) mentioned the occurrence of a leaf ligule in a species of *Anarthria* but did not name the species. To our knowledge, the ligule character has not been used so far in systematic accounts of *Anarthria*, even though it is considered an important taxonomic marker in various groups of Poales (Goetghebeur, 1998; Kellogg, 2015), including the restiid clade (Sokoloff et al., 2015).

Our work was carried out with the aim to assess (i) whether *Anarthria gracilis* in its traditional circumscription is monophyletic and (ii) whether the occurrence of a long and conspicuous leaf ligule can be used in the taxonomy of *Anarthria*. We undertook sampling of all currently recognised species of dithecal restiids from different populations during our field work in Western Australia, together with available herbarium specimens, and subsequently conducted DNA sequencing and morphological observations.

## Materials and Methods

### Field work

Field work in Western Australia for the present study was conducted by CIF, TDM and DDS. We collected specimens of *Anarthria, Lyginia* and *Hopkinsia* in as many localities as possible. Scientific collecting licences and permissions to access reserve land were provided by the Department of Parks and Wildlife, Western Australia, now Department of Biodiversity, Conservation and Attractions (SW016575, SW017203, SW018172, CE006047).

### Morphology and anatomy

PERTH is the primary herbarium for the flora of Western Australia, with representative coverage of all biogeographic regions of the state. As *Anarthria* is endemic to Western Australia, the present study is largely based on collections in PERTH. All relevant specimens in PERTH were investigated with respect to characters traditionally used in the taxonomy of *Anarthria*. Herbarium specimens identified as *Anarthria gracilis* × *humilis* (*Briggs 7471*, *9940B*) together with new gatherings made for this study by CIF and TDM (*Fomichev & Macfarlane WA439*) were examined. Special attention was paid to leaf morphology and the occurrence of a conspicuous ligule. Extensive herbarium collections made by four of us (BGB, CIF, TDM, DDS) were also used. These are deposited in PERTH, NSW and MW. Collections of various European and Australian herbaria were used physically and as on-line images to investigate type material.

For morphological studies of *Anarthria gracilis* s.l. using scanning electron microscopy (SEM) the following specimens were chosen: *Fomichev & Macfarlane WA644*, *WA654*, *WA700* (MW). Fixed material was dissected in 70% ethanol and then transferred to 100% acetone using the following series: 96% ethanol (twice for 30 min), 96% ethanol:100% acetone (1:1 v/v, 30 min), 100% acetone (three times for 30 min). The material was critical point dried in liquid carbon dioxide using a Hitachi HCP-2 critical point dryer (Hitachi, Tokyo, Japan), coated with gold and palladium using a Giko IB-3 ion-coater (Tokyo, Japan), and imaged using a CamScan S-2 (Cambridge Instruments, Cambridge, Great Britain) at the Laboratory of Electron Microscopy at the Biological Faculty of Moscow University. For light microscopy (LM), the widest part of the leaf lamina (above the leaf sheath) was used. The following herbarium material was analysed: *Fomichev & Macfarlane WA408, WA414, WA439, WA642, WA643, WA647, WA648, WA654, WA655, WA659, WA690, WA701, WA730*–MW (Appendix 1). Before sectioning, the material was rehydrated in hot water for two days and transferred into 70% ethanol. Free-hand cross-sections were made and observed in glycerol with an Olympus SZX-4 microscope. Studies of floral morphology were documented using a Carl Ziess stereomicroscope Stemi 2000 fitted with an AxioCam MR digital camera in the following specimens: *Fomichev & Macfarlane WA643*, *WA666*, *WA677*, *WA707*, *WA715*–MW (Appendix 1). Some images were stacked and merged using Adobe Photoshop and Adobe Illustrator (San Jose, CA, USA).

### Taxon sampling for molecular studies

All currently recognised species of dithecal restiids were sampled in the molecular phylogenetic analyses, with special emphasis on the diversity of *Anarthria gracilis*. Samples of studied species were collected in the field or obtained from existing herbarium specimens. Examined taxa, authorities and GenBank accession numbers are given in Appendix 1, together with voucher data for new sequences. *Sporadanthus strictus* (R.Br.) B.G. Briggs & L.A.S. Johnson (Sporadanthoideae) was selected as an outgroup for molecular phylogenetic analyses following the phylogeny of restiids presented by Briggs, Marchant & Perkins (2014).

We studied sequence data from the plastid tRNA-Leu (*trn*L) intron with the *trn*L-*trn*F intergenic spacer (*trn*L-F) and a low copy nuclear gene *at*103 coding Mg-protoporphyrin IX monomethyl ester cyclase (Li et al., 2008).

The chloroplast marker *trn*L-F has already been used in phylogenetic studies of restiids and was found useful in resolving higher-level (Briggs et al., 2000; Eldenäs & Linder, 2000; Hardy, Moline & Linder, 2008; Briggs, Marchant & Perkins, 2010, 2014) as well as lower-level taxonomic problems (Moline & Linder, 2005; Hardy & Linder, 2007; Wagstaff & Clarkson, 2012). Other plastid markers used so far in phylogenetic studies of restiids are the *atp*B-*rbc*L intergenic spacer (Moline & Linder, 2005; Hardy & Linder, 2007; Hardy, Moline & Linder, 2008), the complete *rbc*L gene (Moline & Linder, 2005; Hardy & Linder, 2007; Hardy, Moline & Linder, 2008; Briggs, Marchant & Perkins, 2010, 2014; Wagstaff & Clarkson, 2012), the *mat*K and flanking *trn*K intron (Moline & Linder, 2005; Hardy & Linder, 2007; Hardy, Moline & Linder, 2008; Briggs, Marchant & Perkins, 2010, 2014; Wagstaff & Clarkson, 2012). Our group worked with *rbc*L, *mat*K, *ycf*1 and *trn*L-F, but only the last one was found to be reliable in the context of the present study of species diversity in *Anarthria*, since the other plastid markers were either too conservative or primers designed in earlier studies of other plant groups were not appropriate.

Earlier species-level molecular phylogenetic studies of Australian Restionaceae s.l. did not involve any nuclear markers due to technical problems. Nuclear markers (*topo*6 exon 6-11, *phy*B) have previously been used in restiids at the clade level (Hochbach, Linder & Röser, 2018). During this current study, we tried to use several additional nuclear markers traditionally used in phylogenetic analysis, for example 5S nontranscribed spacer (5S-NTS) of the nuclear ribosomal DNA and *phy*C from the phytochrome gene family, but failed to get good PCR products. All attempts at sequencing the most useful nuclear marker of angiosperm phylogenetics, nrITS, were not successful in Restionaceae. In the present study, we use another nuclear marker, *at*103, which was found to be informative in other plant groups (Harpke et al., 2013; Daïnou et al., 2016; Takawira-Nyenya et al., 2018). It is used here for the first time in restiids and apparently provides the first attempt at assessing nuclear markers in the species-level taxonomy of the group.

### DNA extraction, amplification and sequencing

Total genomic DNA was extracted from silica gel-dried leaf tissue from wild sources and herbarium material using the NucleoSpin Plant isolation kit (Macherey-Nagel, Düren, Germany) following manufacturer instructions. According to the methods of Taberlet, Gielly & Pautou (1991), the plastid *trn*L-F intergenic spacer region was amplified with the following primers: cB49317F-1 and fA50272R-3. The nuclear gene *at*103 was amplified with the following primers following Li et al. (2008): at103-F and at103-R. Encyclo PCR kit (Evrogen, Moscow, Russia) was used for amplification. PCR cycling conditions for *trn*L-F were 96 °C for 3 min, 96 °C for 30 s, 58 °C for 60 s, then 32 cycles of 72 °C for 2 min, with the final extension of 72 °C for 7 min. For *at*103 amplification the following steps were used: 94 °C for 5 min, 94 °C for 30 s, 55 °C for 40 s, then 35 cycles of 72 °C for 60 s, with the final extension of 72 °C for 10 min. PCR products were purified using a DNA cleaning kit (Evrogen, Moscow, Russia) according to the instructions provided by the manufacturer. PCR was carried out using a Thermocycler 3000 (Biometra, Göttingen, Germany). Cleanup Mini (Evrogen, Moscow, Russia) was used to clean the PCR-product. DNA sequencing was carried out using ABI Prism BigDye Terminator v. 3.1 followed by product analysis in an ABI Prism 3100-Avant Genetic Analyzer (Applied Biosystems, Foster City, CA, USA). Both forward and reverse strands were sequenced for all samples.

### Molecular phylogenetic and principal coordinates analyses

Sequences were aligned using MAFFT (Katoh et al., 2002) and then checked manually in BioEdit (Hall, 1999). Phylogenies were reconstructed using *maximum parsimony (MP)* and *Bayesian inference (BI)*. The MP analysis used PAUP* version 4.0b8 (Swofford, 2003) with tree-bisection-reconnection (TBR) branch swapping and equal weighting of characters; gaps were treated as missing data. For each full heuristic search 500 random sequence addition replicates were run and no more than 1,000 shortest trees were saved in each replicate. The MP bootstrap analysis was performed using 5,000 replicates with TBR branch swapping and random addition of taxa and 1,000 most parsimonious trees from each replicate were saved. For assessment of bootstrap support (BS), we considered 85–100% as strong, 75–84% as moderate and less than 75% as weak support (Kress, Prince & Williams, 2002).

Bayesian analysis was conducted using MrBayes version 3.2.6 (Ronquist et al., 2012), after evaluating the best fitting model according to AIC in MrModeltest 2.3 (Nylander, 2004); best models were K80+G for *at*103 and GTR+G for both *trn*L-F separately and the combined analysis of *at*103 and *trn*L-F. The Bayesian analysis used two independent runs of 25 million generations and four chains sampling every 1,000th generation and discarding the first 2,000 (8%) trees as burn-in, the number of discarded trees was determined by Höhna-Sahlin’s ESS-based estimator in VMCMC version 1.0.1 (Ali et al., 2017). The remaining trees were used to construct the majority-rule consensus tree.

The MP and BI analyses were used for the plastid data-set alone and for concatenated plastid and nuclear data. The number of informative sites was relatively low in our nuclear data-set and taken alone did not produce well-resolved phylogenetic trees. Given the relatively low signal and resolution from the nuclear marker a formal congruence analysis was inappropriate. We justify concatenation of plastid and nuclear data by the fact that results of the combined analyses are highly congruent with morphology, and the morphology in this case is strong. For nuclear data alone we performed a principal coordinates analysis implemented in the program PAST 3.14 (Hammer, Harper & Ryan, 2001) to visualise patterns of variation of the nuclear marker (Degtjareva et al., 2013). All unambiguously aligned characters in all available sequences of *Anarthria* species were used. Gower general similarity coefficients were used. Gaps were treated as a fifth character state.

## Results

### Morphology

**Figure 1:**
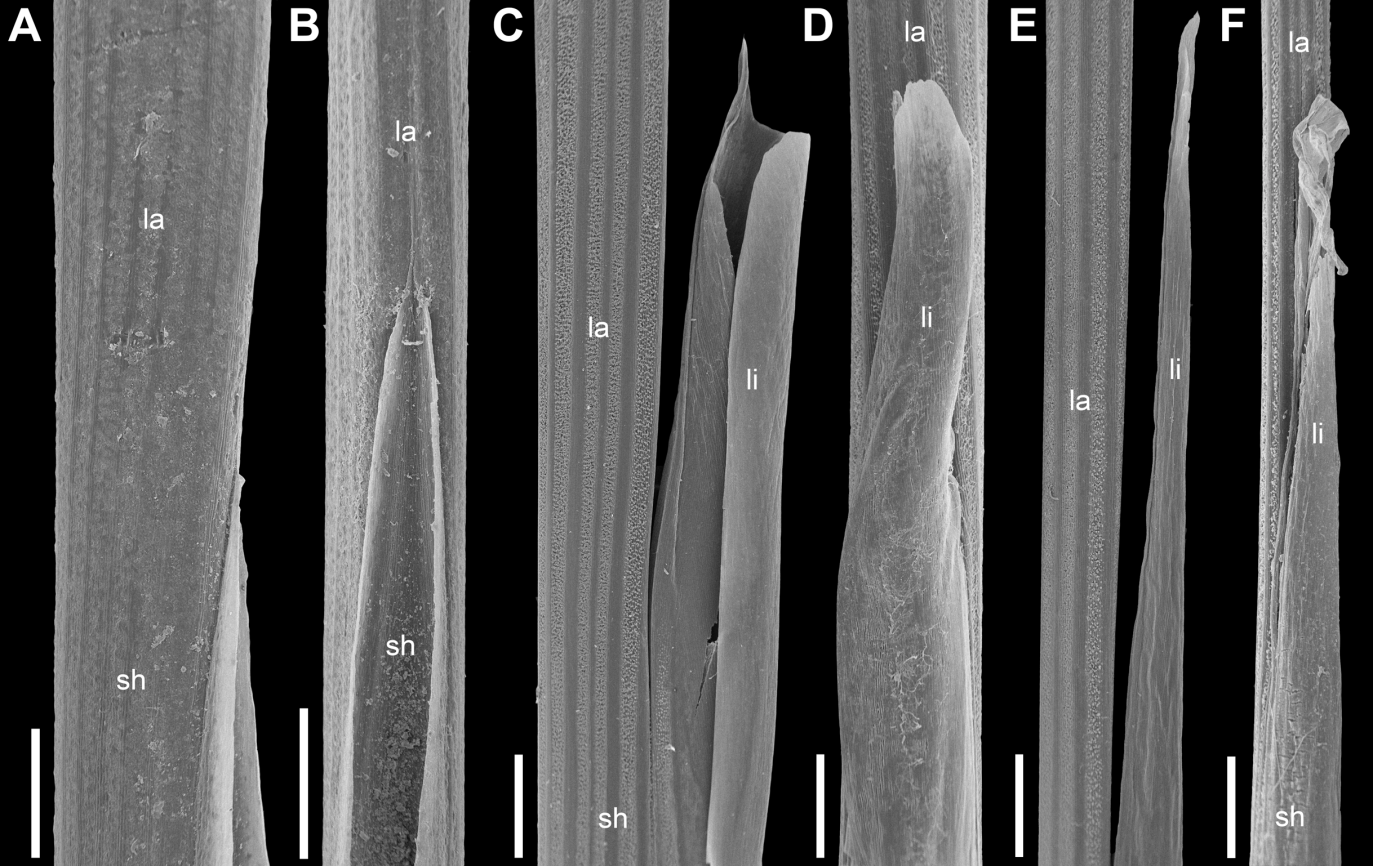
Leaf sheath to lamina transition in plants of the *Anarthria gracilis* complex (SEM). (A and B) *A*. *gracilis* sp. 1 (*A*. *dioica*) *Fomichev & Macfarlane WA654*. (C and D) *A*. *gracilis* sp. 2 (*A*. *gracilis* s.str.) *Fomichev & Macfarlane WA700*. (E and F) *A*. *gracilis* sp. 3 (*A*. *grandiflora*) *Fomichev & Macfarlane WA644*. (A, C and E) side view. (B, D and F) view from the adaxial side. Ligule is very short, barely visible in *A*. *gracilis* sp. 1, but long, conspicuous in *A*. *gracilis* sp. 2 and sp. 3. Abbreviations: la, lamina; li, ligule; sh, sheath. Scales = 1 mm.

**Figure 2:**
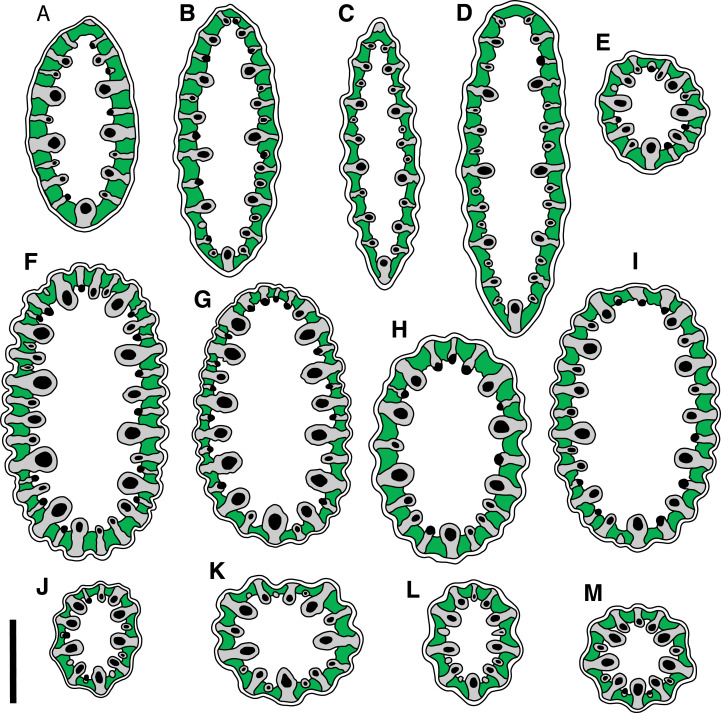
Cross-sections cut in the widest part of leaf lamina (above the leaf sheath) in plants of the *Anarthria gracilis* complex. (A–D) *A*. *gracilis* sp. 1 (*A*. *dioica*). (E) Possible hybrid between *A*. *gracilis* sp. 1 (*A*. *dioica*) and *A. humilis*. (F–I) *A*. *gracilis* sp. 2. (*A*. *gracilis* s.str.). (J–M) *A*. *gracilis* sp. 3 (*A*. *grandiflora*). (A) *Fomichev & Macfarlane WA408*. (B) *Fomichev & Macfarlane WA654*. (C) *Fomichev & Macfarlane WA655*. (D) *Fomichev & Macfarlane WA659*. (E) *Fomichev & Macfarlane WA439*. (F) *Fomichev & Macfarlane WA414*. (G) *Fomichev & Macfarlane WA690*. (H) *Fomichev & Macfarlane WA701*. (I) *Fomichev & Macfarlane WA730*. (J) *Fomichev & Macfarlane WA642*. (K) *Fomichev & Macfarlane WA643*. (L) *Fomichev & Macfarlane WA647*. (M) *Fomichev & Macfarlane WA648*. Green = chlorenchyma; white = epidermis (outer layer) and parenchyma (central region); grey = sclerenchyma; black = vascular bundle. Scale bar common to all images = 600 μm.

**Figure 3:**
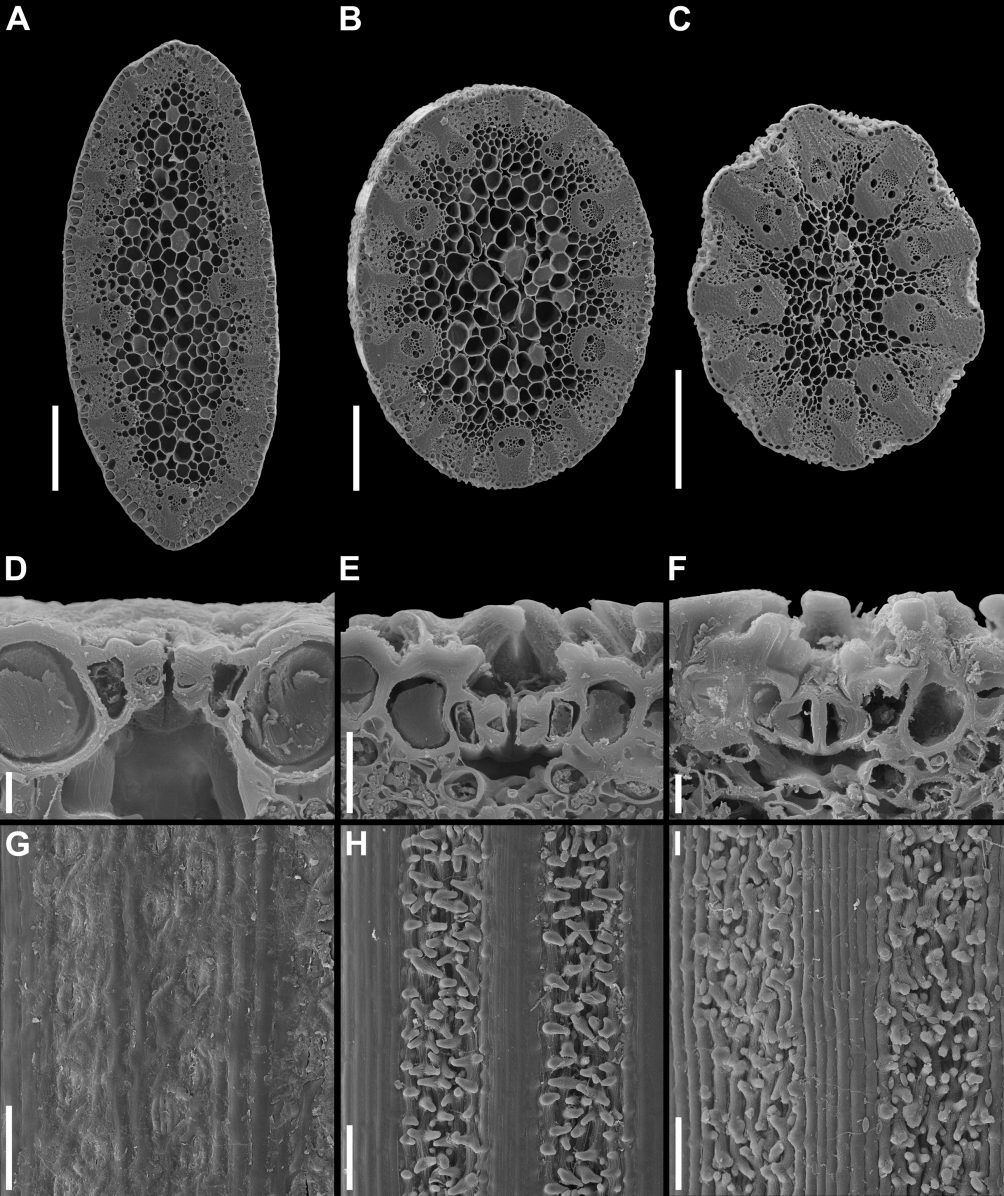
Details of leaf anatomy in *Anarthria gracilis* complex (SEM). (A, D and G) *A*. *gracilis* sp. 1 (*A*. *dioica*) *Fomichev & Macfarlane WA654*. (B, E and H) *A*. *gracilis* sp. 2 (*A*. *gracilis* s.str.) *Fomichev & Macfarlane WA700*. (C, F and I) *A*. *gracilis* sp. 3 (*A*. *grandiflora*) *Fomichev & Macfarlane WA644*. (A–C) Lamina cross-section. (D–F) stomata in cross-section. (G–I) lamina epidermis surface view. Scales: A, B and C = 300 μm, D and F = 10 μm, E = 30 μm, G, I and H = 100 μm.

Specimens of the *Anarthria gracilis* complex examined in the current article differ from each other in details of foliage leaf morphology (Figs. 1–3). Characters revealed during this study allowed distinguishing three groups of *A. gracilis* s.l. that can be seen clearly in herbarium collections, except for some specimens collected without foliage leaves.

In *A. gracilis* sp. 1 the leaf ligule is very short (<0.1 mm at its midpoint), in the form of narrow joining margins of the leaf sheath at the transition to the ensiform lamina (Figs. 1A and 1B). Leaves of *Anarthria gracilis* sp. 2 (Figs. 1C and 1D) and *A*. *gracilis* sp. 3 (Figs. 1E and 1F) possess a long and conspicuous ligule located on the adaxial side in the transition zone between the sheath and lamina. The ligule length is 5–15 mm in *A*. *gracilis* sp. 2 and 5–12 mm in *A*. *gracilis* sp. 3. The ligule is formed as a membranous outgrowth narrowing from the bottom towards the top and with an entire margin. A similar, but shorter (2–2.5 mm long) ligule is present in all specimens of *A. polyphylla*. The ligule condition of *A. gracilis* sp. 1 is shared with that of all other species of *Anarthria* except *A. polyphylla*, *A. gracilis* sp. 2 and 3. Extensive field observations in numerous localities in southwest Western Australia confirmed that the differences in ligule morphology are stable and clearly expressed. In *Lyginia* and *Hopkinsia*, the leaves are reduced to culm sheaths with a short cylindrical appendage that represents the leaf lamina. These two genera possess no ligule. The distal edge of the sheath forms two auricles as outgrowths on both sides of the sheath-to-lamina transition zone in *Hopkinsia*. Such auricles are absent in *Lyginia* and *Anarthria*.

In *A. gracilis* sp. 1 (Figs. 2A–2D and 3A) and *A. gracilis* sp. 2 (Figs. 2F–2I and 3B), leaves possess a laterally flattened lamina. The lamina of *A. gracilis* sp. 2 is less pronouncedly flattened in the vertical plane than in *A. gracilis* sp. 1. It is elliptic in cross-section. In contrast, the leaf lamina of *A*. *gracilis* sp. 3 (Figs. 2J–2M and 3C) is almost circular in cross section, thus the lamina is filiform. The overall size of lamina cross section is considerably smaller in *A. gracilis* sp. 3 than in the two other groups (Fig. 2). Among the other species of *Anarthria*, the leaf lamina is ensiform in *A*. *prolifera*, *A*. *scabra* and *A*. *laevis*, whereas it is filiform in *A*. *humilis* and *A*. *polyphylla*, as well as in all three species of *Lyginia*. *Hopkinsia* differs from the rest of dithecal restiids in its narrow but dorsiventrally flattened leaf lamina.

The three groups of the *A*. *gracilis* complex recognised here are different in leaf epidermis characters (Fig. 3). In ligulate specimens, the epidermis surface forms regular longitudinal stripes of cell rows with smooth outer periclinal cell walls and rows with papillose cells (Figs. 3H and 3I), whereas in the non-ligulate *A*. *gracilis* sp. 1, the whole leaf surface is smooth without papillose stripes (Fig. 3G). In these papillose stripes, the leaf surface is concave in dry leaves (*A*. *gracilis* sp. 2, Figs. 2F–2I, and *A*. *gracilis* sp. 3, Figs. 2J–2M), so that the leaf lamina appears to be slightly ribbed in cross-section (not taking into account the papillae themselves). The ribs are more expressed in *A*. *gracilis* sp. 3 (Fig. 3C). In all studied groups of *A*. *gracilis*, stomata are arranged in a longitudinal linear pattern. In *A*. *gracilis* sp. 1, stomata can be observed in surface view (Figs. 3D and 3G), however in the two other groups, stomata are hidden by the papillae (Figs. 3E, 3F, 3H and 3I).

Although we have not quantified the differences, plants of *A. gracilis* sp. 3 differ from those of the two other groups of *A. gracilis* in being generally smaller, with leaves shorter than in *A*. *gracilis* sp. 2.

**Figure 4:**
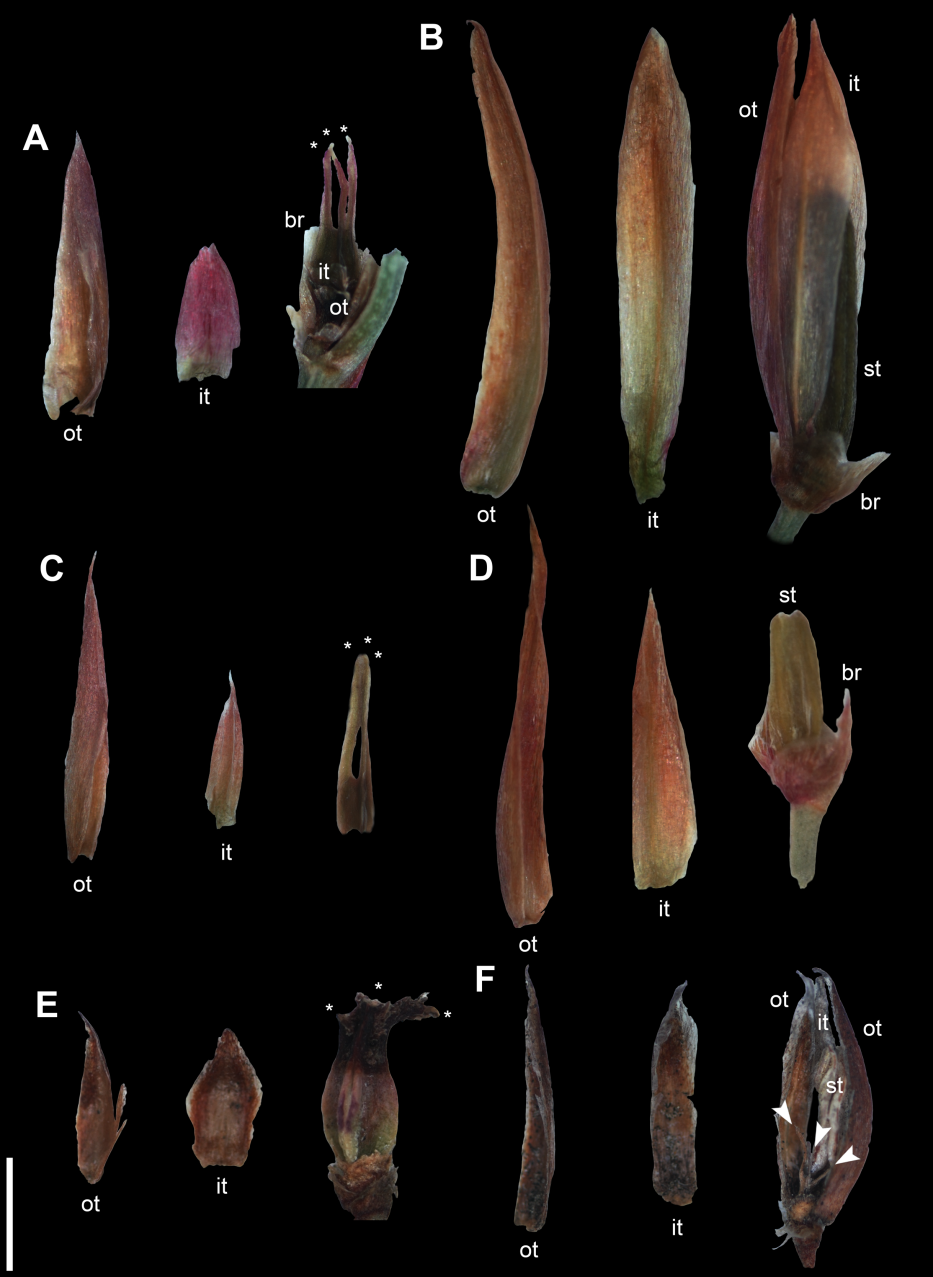
Dissected young flowers of members of the *Anarthria gracilis* complex. (A, C and E) Female flowers. (B, D and F) Male flowers. (A) *A*. *gracilis* sp. 1 (*A*. *dioica*) *Fomichev & Macfarlane WA677*. (B) *A*. *gracilis* sp. 1 (*A*. *dioica*) *Fomichev & Macfarlane WA666*. (C) *A*. *gracilis* sp. 2. (*A*. *gracilis* s.str.) *Fomichev & Macfarlane WA707*. (D) *A*. *gracilis* sp. 2. (*A*. *gracilis* s.str.) *Fomichev & Macfarlane WA715*. (E and F) *A*. *gracilis* sp. 3 (*A*. *grandiflora*) *Fomichev & Macfarlane WA643*. Abbreviations: br, bract; it, inner tepal; ot, outer tepal; st, stamen. Arrow = stigma of pistillode. Asterisk = stigma of fertile gynoecium. Scale bar common to all images = 2 mm.

Species of *Anarthria* differ from each other in some floral characters (Fig. 4). In all three species of the *A*. *gracilis* complex, inner whorl tepals of female flowers are usually shorter than the outer whorl tepals (Figs. 4A, 4C and 4E). The inner tepals of female flowers are shorter than the outer ones in *A*. *humilis*, *A*. *prolifera* and *A*. *scabra* as well, but are of about the same length in *A*. *laevis* and *A*. *polyphylla*. In male flowers of *A*. *gracilis* sp. 1 and *A*. *gracilis* sp. 2 tepals of both whorls are often of about the same length, whereas the inner tepals are shorter than the outer tepals in male flowers of *A*. *gracilis* sp. 3. Tepals are all of equal length or the outer tepals slightly longer that the inner tepals in male flowers of *A*. *prolifera*, *A*. *scabra*, *A*. *laevis* and *A*. *polyphylla*. The outer tepals of *A*. *humilis* are longer than the inner tepals. In *Hopkinsia*, both male and female flowers have the inner tepals longer than the outer tepals. Some, but not all male plants of *A*. *gracilis* sp. 3 examined possessed a sterile gynoecium (pistillode) (Fig. 4F).

Our analysis of herbarium collections and observations in nature confirmed that the traditionally recognised species of *Anarthria* other than *A. gracilis* are morphologically well-defined and recognisable using diagnostic characters used in the literature. The only exception is the occurrence of a very few specimens that share morphological characters of *A. humilis* and *A. gracilis* sp. 1. Two samples of this form (*Briggs 9940B, 7471*, PERTH, NSW) were collected prior to this study in the NW part of the range of *A. humilis*, where the species co-occurs with *A. gracilis* sp. 1. These plants agree with typical *A. humilis* in all characters including filiform leaf lamina (Fig. 2E) and inflorescences with as few as 3–5 flowers except in having longer leaf laminae (about 7–18 mm). In the latter character, they resemble *A. gracilis* sp. 1. Based on morphology, the specimens *Briggs 9940B* and *7471* were identified by the collector as *A*. *gracilis* × *humilis*. Subsequent field work in one of these localities in another season resulted in another sample of this type (*Fomichev & Macfarlane WA439*, MW). Note that *A. gracilis* sp. 1 and *A. humilis* share the occurrence of extremely short leaf ligules. The same condition is found in *Briggs 9940B, 7471* and *Fomichev & Macfarlane WA439*.

### Plastid *trn*L-F region analysis

**Figure 5:**
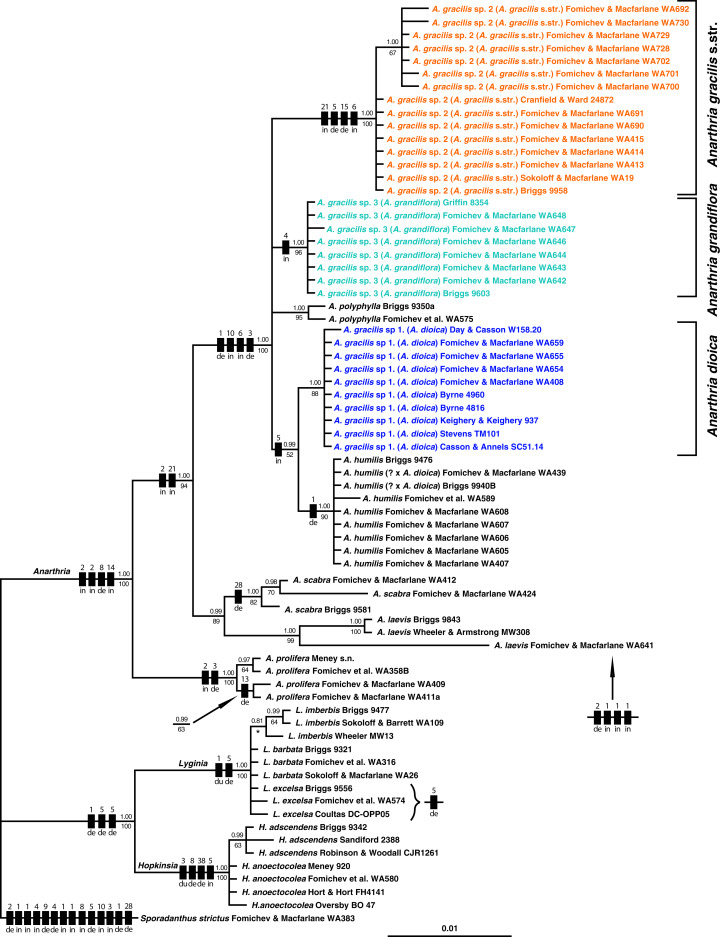
Bayesian tree of dithecal restiids based on the plastid marker *trn*L-F. Numbers above and below branches indicate respectively Bayesian posterior probabilities (PP) and bootstrap support values (BS) found in maximum parsimony analysis (MP). Bars on branches indicate inferred indel events. Numbers above bars correspond to indel length (bp). Abbreviations below boxes indicate the inferred indel type: de, deletion, du, duplication; in, insertion. Asterisk = the inferred clade has no bootstrap support in MP tree.

The *trn*L-F data set comprised 71 samples with 857 positions in the alignment, of which 105 characters are parsimony-informative (Table 1). Topologies of the BI and MP trees were congruent with each other, and both analyses are summarised in Fig. 5. Monophyly of all three genera of dithecal restiids is well-supported. Relationships within *Lyginia* and *Hopkinsia* are partly unresolved. All species of *Anarthria* except *A. gracilis* are found to be monophyletic. *Anarthria prolifera* is well-supported as a sister group to the rest of *Anarthria*. *Anarthria scabra* R.Br. forms a clade with *A. laevis* R.Br. (PP 0.99, BS 89%). Samples examined in the phylogenetic analysis fitting the traditional view on limits of *A. gracilis* are segregated into three well-supported (PP 1.00, BS 88–100%) groups recognised here as sp. 1, sp. 2 and sp. 3 according to the morphological characters outlined above (Fig. 5). Relationships of the specimens traditionally assigned to *A. gracilis* can be summarised as *A. polyphylla* Nees + *A. gracilis* sp. 2 + *A. gracilis* sp. 3. + [*A. humilis* Nees *+ A. gracilis* sp. 1]. All five groups listed above form a well-supported clade. *Anarthria gracilis* sp. 1 unites with *Anarthria humilis* with PP 0.99 and BS 52%. The samples morphologically intermediate between *A. humilis* and *A. gracilis* sp. 1. (*Briggs 9940B* and *Fomichev & Macfarlane WA439*) are robustly placed in the clade of *A*. *humilis*.

**Table 1:** Information on DNA markers and characteristics of maximum parsimony phylogenetic analysis.

	*trn*L-F	*at*103	*trn*L-F and *at*103 combined data
Total accessions	71	50	44
Total of aligned characters	857	236	1093
Constant characters	634	181	838
Pasimony-informative characters	105	33	112
Parsimony-uniformative variable characters	118	22	143
Length of the shortest trees	268	90	331
Retained trees	30	493,000	241,000
CI/RI of the shortest trees	0.85/0.98	0.65/0.89	0.75/0.93

### Nuclear *at*103 region and combined data set analyses

**Figure 6:**
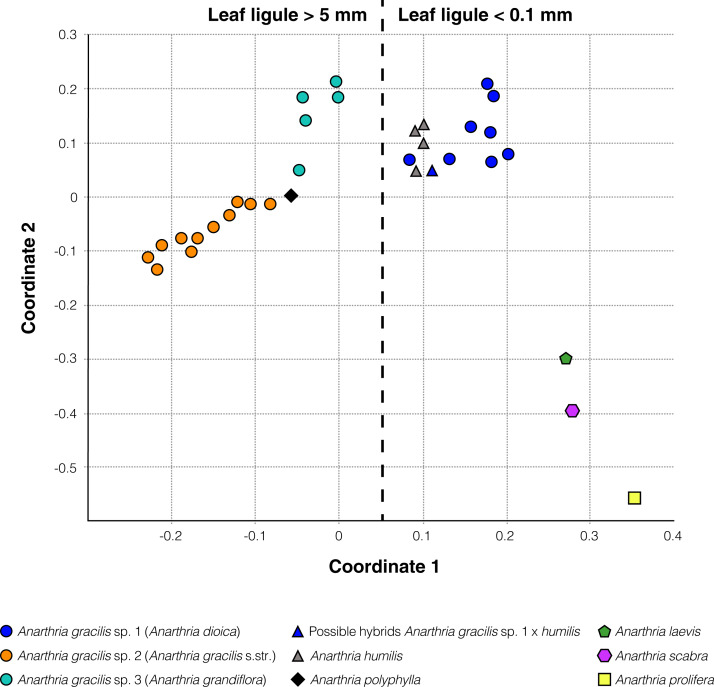
Principal coordinates analysis of *at*103 sequence data for examined samples of *Anarthria* species. Accessions with very short, barely visible and long, conspicuous leaf ligule form two well-separated groups. The three groups segregated from *A. gracilis* are coloured in the same way as in Fig. 5.

**Figure 7:**
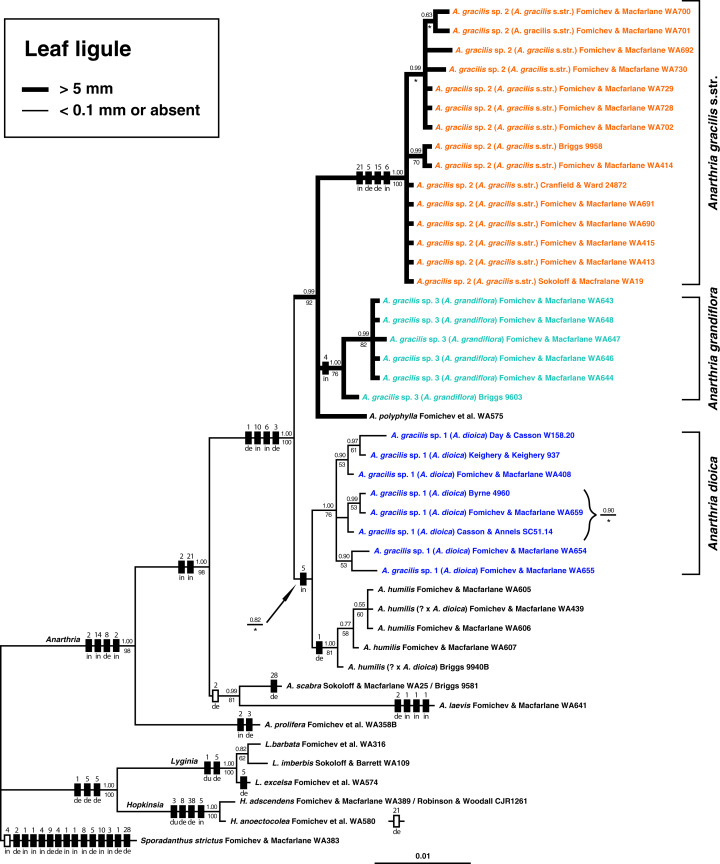
Bayesian tree inferred from the analysis of concatenated nuclear (*at*103) and plastid (*trn*L-F) datasets. Numbers above and below branches indicate Bayesian posterior probabilities (PP) and bootstrap support values (BS) found in maximum parsimony analysis (MP), respectively. Boxes on branches indicate inferred indel events. Black box = plastid indel, white box = nuclear indel. Numbers above boxes correspond to indel length (bp). Abbreviations below boxes indicate the inferred indel type: de, deletion; du, duplication; in, insertion. Asterisk = the inferred clade has no bootstrap support in MP tree. Branch thickness: thick = leaf ligule long, conspicuous, more than 5 mm long; thin = leaf ligule very short, barely visible, less than 0.1 mm long or absent.

Alignment of the nuclear marker *at*103 included 50 samples with 236 bp, among which 33 were parsimony-informative (Table 1). The principal coordinates analysis (Fig. 6) showed a clear division into pronouncedly ligulate (*A*. *polyphylla*, *A*. *gracilis* sp. 2 and sp. 3) and those with ligules barely visible (*A*. *humilis*, *A*. *gracilis* sp. 1, *A*. *laevis*, *A*. *polyphylla*, *A*. *prolifera*, *A*. *scabra*). Phylogenetic trees obtained using this marker alone are not well resolved, but analysis of a combined data set of *at*103 and *trn*L-F provided additional information (Fig. 7).

The combined analysis involved 44 samples for which sequences of both markers were available. Out of the total of 1,093 bp, 112 were parsimony-informative (Table 1). The results of the combined analysis are close to those based on *trn*L-F alone. Monophyly of all three genera, a clade of *Lyginia* + *Hopkinsia* and the basal position of *A. prolifera* in *Anarthria* are well-supported. *Anarthria scabra* and *A. laevis* form a sister clade to a clade comprising all examined samples of *A. gracilis* s.l., *A. humilis* and *A. polyphylla*. Each of the three groups of *A. gracilis* is well-supported (PP 1.00, BS 76–100%). *Anarthria gracilis* sp. 1 shows a close affinity to *A. humilis* in the Bayesian tree (0.82 PP), however the MP analysis did not reveal this relationship. A clade comprising *A. gracilis* sp. 2, *A. gracilis* sp. 3 and *A. polyphylla* is highly supported (PP 0.99, BS 92%). As in the tree inferred from the plastid marker alone, the samples *Briggs 9940B* and *Fomichev & Macfarlane WA439* that combine morphological features of *A. gracilis* sp. 1 and *A. humilis* are placed within the *Anarthria humilis* clade.

### Phylogenetic significance of indels

Indels were mapped on the branches of the phylogenetic trees resulting from our phylogenetic analyses (Figs. 5 and 7). The plastid region *trn*L-F showed more abundant and far wider length variations than the nuclear marker *at*103. The distribution of indels supports our tree topologies.

## Discussion

### Comparison of the results inferred from analyses of nuclear and plastid DNA markers

The molecular phylogenetic study of the genus *Anarthria* was carried out using two markers. The first set of trees obtained is based on the plastid marker *trn*L-F, wherein several specimens for each species were examined (Fig. 5). The traditionally accepted species *A. gracilis* is divided into three groups, which strongly differ morphologically from each other. The sampling of several specimens for each species allowed the monophyly of the other traditionally accepted species within *Anarthria* and closely related genera *Lyginia* and *Hopkinsia* to be estimated. Each species of *Anarthria* except *A. gracilis* formed a monophyletic group with maximum posterior probability in BI and with high to moderate level of bootstrap-support in the MP analysis. Significant differences in nucleotide sequences between specimens are revealed within *A*. *scabra*, *A*. *laevis* and *A*. *prolifera*. These differences should be explored in further phylogeographic studies. In contrast to species of *Anarthria*, the traditionally accepted species of *Lyginia* and *Hopkinsia* remain unresolved in the *trn*L-F tree.

This is the first time that a nuclear marker has been used in Restionaceae to clarify species limits. Taken alone, our nuclear marker did not produce well-resolved trees, but its use in combination with *trn*L-F revealed a clade that includes all species of *Anarthria* with conspicuous ligules. Relationships of pronouncedly ligulate species of *Anarthria* were unresolved in plastid trees, so the addition of the nuclear marker resulted in a more resolved topology that is congruent with our morphological findings.

### Leaf ligule as an important diagnostic morphological character in *Anarthria*

The ligule represents a membranous outgrowth on the adaxial side of the leaf in the region between the sheath and lamina or sheath and petiole if the latter is present and occurs in many groups of monocots such as Alismatales and commelinids, most notably in Poales and Zingiberales (Philipson, 1935; Rudall & Buzgo, 2002). In grasses, the ligule forms a membranous appendage or transversal row of hairs in the sheath-to-lamina transition zone and plays an important role in identification, not only at the generic level but also in distinguishing species in some genera. In restiids, a ligule occurs in some taxa of both monothecal and dithecal clades (Cutler, 1969; Linder & Caddick, 2001; Sokoloff et al., 2015; Briggs, Connelly & Krauss, 2020a; this study). Data on ligule distribution among monothecal Restionaceae as well as its morphology are scarce, but preliminary results of an investigation of this feature using herbarium material with subsequent character-mapping revealed a high level of homoplasy within the restiid clade and stability at the species level (Fomichev, 2016). A detailed study of leaf diversity, development and evolution across Restionaceae s.l. (and apparently some other Poales) is needed and may reveal new useful taxonomic characters. The presence of a ligule was noted in the literature (Cutler, 1969; Briggs, Connelly & Krauss, 2020a) for one species of *Anarthria*, without clarification of which species it was. According to our observations, a conspicuous ligule is present in three species of dithecal restiids: *Anarthria gracilis* sp. 2, *A*. *gracilis* sp. 3 and *A*. *polyphylla* (Fig. 7). All three species formed a clade in Bayesian and MP trees based on combined data of both plastid and nuclear markers. These data suggest that a conspicuous ligule appeared once in the course of evolution of dithecal restiids, independently from the ligules of monothecal restiids.

*Anarthria gracilis* sp. 1, characterised by equitant leaves (Figs. 2A–2D and 3A) with an extremely short, barely visible ligule (Figs. 1A and 1B), occupies a position sister to *A*. *humilis* (that has the same ligule morphology) in our phylogenetic trees (Figs. 5 and 7). An earlier phylogenetic study suggested a clade comprising *A*. *humilis* and *A*. *polyphylla* (Briggs, Marchant & Perkins, 2014). We believe that this difference from our tree topology is due to their much less representative taxon sampling.

### Species limits in the *Anarthria gracilis* complex

Our data support the recognition of eight (rather than six) species of *Anarthria*, with material currently classified as *A. gracilis* (Meney & Pate, 1999; Briggs, Connelly & Krauss, 2020b) belonging to three distinct species referred above as to sp. 1, sp. 2 and sp. 3. Molecular studies focused on cryptic species have revealed their occurrence in many plant groups (Ito et al., 2019; Sokoloff et al., 2019; Telford et al., 2019). Even though the three species recognised here in the *A. gracilis* complex appear to be superficially similar, careful morphological analysis revealed clear and useful diagnostic characters. Therefore, the three species cannot be regarded as cryptic. It is remarkable that by 1839 (the first collection of *A. gracilis* sp. 3) all three species had already been collected.

Analysis of type material revealed that the name *A. gracilis* R.Br. s.str. should be used for our *A. gracilis* sp. 2. Nees von Esenbeck (1841, 1846) described three more species in the group: *A. grandiflora* Nees, *A. canaliculata* Nees and *A. ischaemoides* Nees. Based on analysis of type material, we identify *A. canaliculata* Nees and *A. ischaemoides* Nees as synonyms of *A. gracilis*, whereas *A. grandiflora* Nees can be used to name one of the two segregate species (*A*. *gracilis* sp. 3). This species is morphologically close to *A. gracilis* s.str. but differs mainly in width and cross-section shape of the leaf lamina. We only paid attention to these differences after getting our first molecular phylogenetic data, but later found that the two species can be easily separated from each other in herbarium collections. While describing *A. grandiflora*, Nees von Esenbeck (1841) provided a short description, but made no comparison with any other species of the genus, including *A. gracilis*. Another species newly described on the same page, also without any comparisons, is *A. humilis* (Nees von Esenbeck, 1841). Diagnostic characters are not clear from these descriptions and *A. humilis* is wrongly described as having one rather than two ‘bracteoles’ (=bracts of reduced spikelets). When describing *A. canaliculata* and *A. ischaemoides* based on Preiss collections, Nees von Esenbeck (1846) indicated that the former differs from *A. gracilis* in plants being taller, flowers twice as long and in deeply canaliculate leaves. Plant height varies in *A. gracilis* s.str. and the occurrence of longitudinal grooves is not unusual in this species. As for the difference in flower length, Nees von Esenbeck (1846) based his description of *A. canaliculata* on male plants only. Male flowers of *A. gracilis* are longer than female flowers. According to Nees von Esenbeck (1846), diagnostic characters of *A. ischaemoides* are more rigid and wider culms and much larger female flowers. Type material indeed has wide culms, but female flowers are of ordinary length. These plants fit our interpretation of *A. gracilis* s.str. Bentham (1878) placed *A. grandiflora, A. canaliculata*, *A. ischaemoides* and *A. humilis* in synonymy of *A. gracilis*. His view on *A. humilis* was likely caused by the fact that Nees von Esenbeck (1841) did not provide its clear characters. Also, Bentham apparently did not see original material of *A. humilis*. We support subsequent accounts recognising *A. humilis* as distinct from *A. gracilis* (Green, 1981; Rye, 1987; Meney & Pate, 1999; Briggs, Connelly & Krauss, 2020a, 2020b; apparently, this view was already adopted by Hieronymus (1889)). Among other characters, it differs from *A. gracilis* s.str. in the absence of a conspicuous long ligule.

The species recognised above as *A. gracilis* sp. 1 had been collected as far back as in 1826 by the famous French explorer J.S.C. Dumont d’Urville (1790–1842) during the first voyage of the Astrolabe that culminated in identification of the site of the shipwreck of La Pérouse. As Dumont d’Urville collected only male plants, finding proper taxonomic placement of the material was problematic. The material was investigated by Steudel (1855) who wrongly identified it as a member of *Juncus* in another family, Juncaceae. As members of Juncaceae normally have bisexual flowers, the name *Juncus dioicus* Steud. was proposed (Steudel, 1855). It is not surprising that Steudel did not compare his new species with *Anarthria gracilis* R.Br. that was already described by this time (Brown, 1810). The present study provides morphological as well as molecular evidence for recognising this species, including conspicuous differences in ligules. The name is transferred here to *Anarthria* as *A. dioica* (Steud.) C.I. Fomichev.

### Possible interspecific hybridisation in *Anarthria*

**Figure 8:**
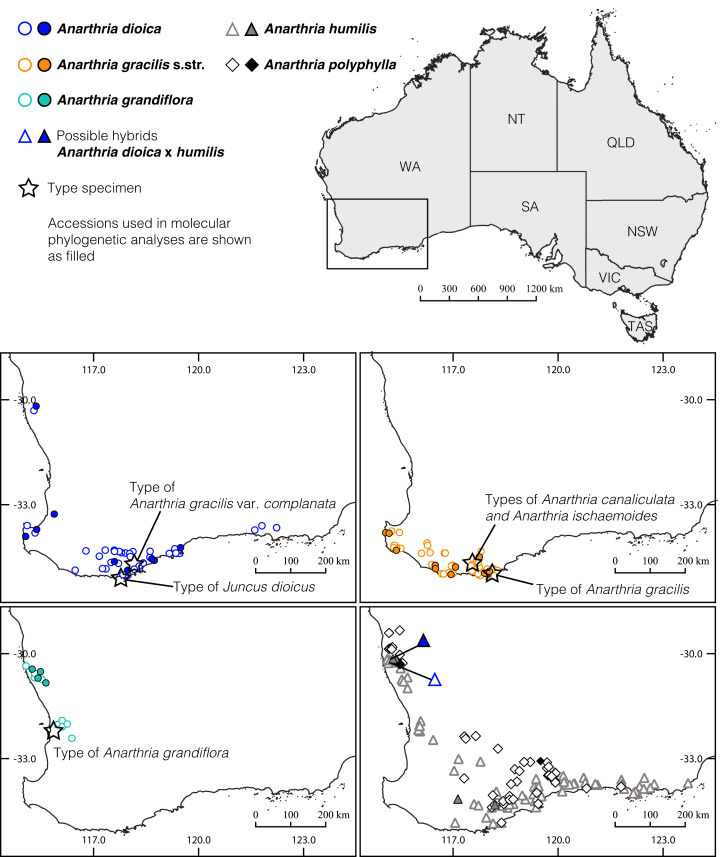
Distribution of the members of the *Anarthria gracilis* complex and the related species *A*. *humilis* and *A*. *polyphylla* in southwest of Western Australia. Locality records of specimens are provided by Western Australian Herbarium (PERTH). Taxon labelling follows that in Fig. 6. Accessions of *Anarthria gracilis* complex used in molecular phylogenetic analysis are shown as filled figures of the corresponding colour for each group.

*Anarthria humilis* clearly differs from *A*. *dioica* in terete (rather than ensiform) leaves, very narrow (0.5–1 mm wide) culms and few-flowered inflorescences. In addition, plants of *A. humilis* are much smaller than those of *A. dioica*. Three examined specimens from two localities near Mount Lesueur fit all important characters of *A. humilis* but differ from the latter in attaining greater plant height. In this character, they resemble *A. dioica*. Typical *A. humilis* as well as *A. dioica* also occur in this area (Fig. 8). It has been suggested that *A*. *gracilis* may hybridise with *A*. *humilis* (Briggs, Connelly & Krauss, 2020b). We were able to produce molecular data for two of these problematic specimens (*Briggs 9940B* and *Fomichev & Macfarlane WA439*). The plastid marker clearly places both specimens in *Anarthria humilis* (Figs. 5 and 7). Our nuclear marker is not informative enough to confirm or reject the hypothesis of hybridisation between *A*. *dioica* and *A*. *humilis*. A fine-scale study including extensive localised sampling and NGS analysis is warranted to test this hypothesis. If present, such hybridisation appears to be localised in a narrow area near the north-western limit of distribution ranges of both species. The ranges of *A*. *dioica* and *A*. *humilis* also overlap extensively along the southern coast of the southwest of Western Australia (Fig. 8), but intermediate specimens are unknown there. Possible occurrence of introgression between *A*. *dioica* and *A*. *humilis* does not make problematic our main conclusion of taxonomic resolution of *A. gracilis* s.l. into three separate species. The overall morphological and molecular diversity of the material traditionally classified as *A. gracilis* s.l. can by no means be explained by possible introgression with *A. humilis*. Morphologically, *A. dioica* cannot be interpreted as ‘intermediate’ between *A. humilis* and the two long-ligulate species *A. gracilis* s.str. and *A. grandiflora*. For example, it has the most pronouncedly ensiform shape of leaf lamina in the whole group (Fig. 2).

### Taxonomic treatment

Images of type specimens marked as “!” have been seen by CIF, TDM, BGB and DDS. Except for those in LE, the specimens were also previously examined in the respective herbaria by BGB. All material from LE was examined by CIF and DDS. All material from PERTH was examined by CIF and TDM and specimens from NSW were examined by BGB.

***Anarthria gracilis* R.Br.**, *Prodromus Florae Novae Hollandiae et Insulae Van-Diemen 1:* 249 (1810). Lectotype (designated by B.G. Briggs in Mabberley & Moore, Regnum Vegetabile 160, in press): King George IIId Sd. [King Georges Sound], [W.A.], *R. Brown (Bennett 5841)*, BM 000991239!; isolectotypes: BM 000991240!, E 00346011!, K 001056262!, MEL 14501 = *Anarthria gracilis* sp. 2 of our Results section.

Taxonomic synonyms:

*Anarthria canaliculata* Nees in J.G.C. Lehmann, *Plantae Preissianae* 2: 62 (1846). Holotype: in subturfosis hieme inundatis planitiei prope oppidulum “Albany” (Plantagenet), 29. Jan. [18]41, *Preiss 1803*, LD 1354037!, isotypes LE 01076952!, LE 01076945 (extreme left plant only)!

*Anarthria ischaemoides* Nees in J.G.C. Lehmann, *Plantae Preissianae* 2: 62 (1846); *Anarthria gracilis* R.Br. var. *ischaemoides* (Nees) Domin, *Journal of the Linnean Society, Botany* 41: 268 (1912). Original material: in solo turfoso inter frutices planitiei prope urbeculam “Albany” (Plantagenet), 5 Oct. [18]40, *Preiss 1815*, LD 1746884!, MEL 14541, P 00748637!, LE 01076946!, LE 01076945 (two plants in the bottom right part of the sheet)!; ad Stirling’s terrace, Plantagenet, W.A., 23 Sept. 1840, *Preiss 1816*, LD 1312106!, LE 01076947! Lectotype (designated here): LD 1746884.

Note: specimens LE 01076946 and LE 01076945 are heterogeneous and contain plants that we identify as *A. gracilis* s.str. and *A. dioica*. LE 01076945 has labels of *Preiss 1815* and *Preiss 1803*, but the plants of *A. dioica* appear to be added from a third, unlabeled collection. They are mounted in the middle upper part of the herbarium sheet far from either of the two labels. LE 01076946 may be a similar mixture.

Herb, evergreen, dioecious, perennial, caespitose, forming tufts or large tussocks. Rhizomes short, ascending, covered by weathered leaf bases. Leaves basal, linear, 15–75 cm long; sheathing base 5–10 cm long, pale purple to brown; lamina laterally compressed, 1–2 mm wide dorsiventrally, flexible or rigid, striate, acuminate. Ligule present, 5–15 mm long, 2–3.5 mm wide, membranous, appressed to the next leaf or stem, margins overlapping, at least at the base. Flowering stem of a single internode, arising from the base, erect, semi-terete, 10–80 cm long, 1–2.5 mm wide, striate. Inflorescence 25–85 cm tall, of reduced spikelets each with 1–2 bracts; spikelets sessile or on a short peduncle; a single flower in the axil of the lowermost bract. Male inflorescences 4.5–10 cm long with 20–40 spikelets, female inflorescence 2–8 cm long with 10–45 spikelets. Inflorescence branches short, erect or more rarely contorted, papillate. Bracts subtending flowers deltoid, 1.5–2.5 mm long, acute, broad, membranous. Spathe subtending inflorescence caducous, linear-lanceolate, 6.5–20 cm long, 3–8 mm broad, purple to brown, scarious, lamina short to prominent, sometimes with a ligule. Male flowers: tepals 6, narrow-lanceolate, acute or acuminate; outer tepals 5.5–7 mm long, keeled; inner tepals 4.5–7 mm long, keeled; filaments 1.5–6 mm long; anthers 2–4 mm long, exserted at anthesis. Pistillode absent. Female flowers: tepals 6; outer tepals narrow-lanceolate, acute, 4–6 mm long, keeled or flat; inner tepals narrow-lanceolate, acuminate, 3–4 mm long, flat; style branches longer than both perianth whorls, exserted laterally between the tips of the outer tepals at anthesis, recurved or straight. Fruit a dry capsule, 3.5–4.5 mm long, 1.5–3 mm broad.

Distribution (Fig. 8): Southern region of Western Australia along the coast-line from the Busselton area to Albany.

***Anarthria grandiflora* Nees**, *Annals and Magazine of Natural History* 6: 50 (1841). Lectotype (here designated): Swan River, W.A., 1839, *Drummond*, CGE05073!; residual syntype: B 100278880! = *Anarthria gracilis* sp. 3 of our Results section. Lectotypification is provided because there are two specimens. The specimen at CGE, which was originally in Lindley’s herbarium, is chosen as lectotype because Lindley made available the specimens for and sponsored publication of Nees’ paper and Nees (1846) subsequently cited the material for the species as “Drummond in Herb. Lindl.”, possibly indicating his view that the main set used for the paper is that now at CGE. Both the CGE and B specimens bear annotations by Nees.

In contrast to some later collections of Drummond for which there is often uncertainty about the collecting locations, there is no doubt that the type material of *A. grandiflora* was collected in the Perth region. This collection by Drummond, dated 1839 in CGE and received in Europe in time to be seen by Nees von Esenbeck for his 1841 publication, was collected before Drummond travelled south of Perth for the first time in late 1840 (Erickson, 1969).

Herb, evergreen, dioecious, perennial, caespitose, forming tufts or tussocks. Rhizomes short, ascending, covered by weathered leaf bases. Leaves basal, linear, 12–36 cm long; sheathing base 3–5 cm long, green to pale purple; lamina terete or subterete, rigid, 0.5–0.6 mm wide dorsiventrally, coarsely striate, acuminate. Ligule present, 5–12 mm long, 2–3.5 mm wide, membranous, appressed to the next leaf or stem, margins overlapping. Flowering stem of a single internode, arising from the base, erect, terete, 17–44 cm long, 0.6–1.2 mm wide, coarsely striate. Inflorescence 14–60 cm tall, of reduced spikelets each with 1–2 bracts; spikelets sessile or on the short peduncle; a single flower in the axil of the lowermost bract. Male inflorescences 2–7 cm long with 7–34 spikelets; female inflorescences 2–3.5 cm long with 7–25 spikelets. Inflorescence branches short, erect, papillate. Bracts subtending flowers deltoid, 1.5–3 mm long, acute, broad, membranous. Spathes subtending inflorescence caducous, linear lanceolate, 7–12.5 cm long, 3–4 mm broad, brown to greenish, scarious to herbaceous, lamina short to prominent, with a ligule. Male flowers: tepals 6, narrow-lanceolate, scarious-membranous, acute or acuminate; outer tepals 4.5–9 mm long, keeled; inner tepals 3.5–5.5 mm long, flat; filaments c. 1.5 mm long; anthers 2.5–3.8 mm long, inserted. Pistillode present or absent in male flowers. Female flowers: tepals 6; outer tepals narrow-lanceolate, acute, 3.2–7 mm long, keeled; inner tepals ovate, obtuse, 2.5–5 mm long, flat; style branches longer than tepals, exserted laterally. Fruit a dry capsule 3.5 mm long, 3 mm broad.

Distribution (Fig. 8): the species is known from thirteen locations in Western Australia including eight in the Jurien Bay area and five in the Perth region.

***Anarthria dioica* (Steud.) C.I. Fomichev, comb. nov.**

Basionym: *Juncus dioicus* Steud., *Synopsis plantarum glumacearum* 2: 309 (1855). Type: Port du Roi Georges, N. Holl., [W.A.], 1826, *[d’]Urville*, *♂*, lectotype (designated here): P 00748635!; residual syntypes: P 00748634!, P 00748636! = *Anarthria gracilis* sp. 1 of our Results section. Steudel annotated all three specimens with an initial identification but wrote the name of his new species only on the the specimen that we designate as the lectotype.

Taxonomic synonym:

*Anarthria gracilis* R.Br. var. *complanata* Domin, *Journal of the Linnean Society, Botany* 41: 267 (1912). Type: Sand plains about Warrungup, W.A., 1910, *A. Dorrien-Smith*, K!

Herb, evergreen, dioecious, perennial, caespitose, forming tufts or tussocks. Rhizomes short, ascending, covered by weathered leaf bases. Leaves basal, linear, 13–39 cm long, sheathing base 2–11 cm long, light green or purple to brown; lamina ensiform, flexible or rigid, 1–1.6 mm wide dorsiventrally, smooth, acuminate. Ligule very short, in the form of narrow joining margins of the leaf sheath (less than 0.1 mm long at its midpoint). Flowering stem of a single internode, arising from the base, erect, flattened, 9–44 cm long, 0.7–1.2 mm wide, smooth. Inflorescences 22–50 cm tall, of reduced spikelets each with 1–2 bracts; spikelets sessile or on a short peduncle; a single flower in the axil of the lowermost bract. Male inflorescence 2–7.2 cm long with 8–42 spikelets, female inflorescence 2–10.5 cm long with 7–21 spikelets. Inflorescence branches short, erect or more rarely contorted, smooth. Bracts subtending flowers deltoid, 1.3–1.8 mm long, obtuse, broad, membranous. Spathe subtending inflorescence caducous, linear-lanceolate, 5–17 cm long, 5–8 mm broad, purple or light green to brown, scarious, lamina short to prominent, without ligule. Male flowers: tepals 6, narrow-lanceolate, acute or acuminate; outer tepals 6–8.5 mm long, keeled; inner tepals 5–8.5 mm long, flat; filaments 1.4–2.1 mm long; anthers 2–4.5 mm long, exserted. Pistillode absent. Female flowers: tepals 6; outer tepals narrow-lanceolate, acute, 2–4.5 mm long, keeled; inner tepals ovate, obtuse, 2–3 mm long, flat; style branches longer than the tepals, recurved or erect, exserted. Fruit a dry capsule 1.8–2.1 mm long, 1.7–2.1 mm broad.

Distribution (Fig. 8): Specimens of *Anarthria dioica* have been found in four disjunct areas of Western Australia: (i) two populations along the coast of the Midwest Region in the Jurien Bay area, (ii) five populations near Busselton, (iii) west of the Warren Region and eastern area of South Coast Region (this includes the vast majority of records) and (iv) three populations north of Esperance.

### A revised key to the species of *Anarthria*

The key is modified from Meney & Pate (1999) and Briggs, Connelly & Krauss (2020a) to include the three species of the *A. gracilis* complex.

1. Leaf ligule present and conspicuous (>2 mm long); leaf surface with alternating longitudinal papillate and non-papillate stripes2
1: Leaf ligule very short (<0.1 mm at its midpoint), barely visible; leaf surface smooth, without papillate stripes4
2. Ligule short, about 2–2.5 mm; plants small, 5–15 cm high; shoot mostly supported above ground on stilt-like roots A. polyphylla
2: Ligule long, about 5–15 mm; plants large, more than 15 cm; roots underground 3
3. Leaf lamina flattened, elliptic in cross-section, with thick rounded edges, in its widest part 1–2 mm wide dorsiventrally (i.e. from the side of sheath opening to opposite side)*A. gracilis* s.str. (=*A*. *gracilis* sp. 2)
3: Leaf lamina filiform, circular in cross-section, usually 0.5–0.6 mm wide*A. grandiflora* (=*A*. *gracilis* sp. 3)
4. Above-ground shoot system extensively branched; spikelets terminal on axes of different orders, but some other branches continue vegetative growth*A*. *prolifera*
4: Leaves and unbranched flowering stems borne directly on underground rhizomes, the vegetative part and inflorescence zone clearly distinct5
5. Leaves 4.5–8(–10) mm wide dorsiventrally; culm and leaf margins coarsely or minutely scabrid*A*. *scabra*
5: Leaves less than 6 mm wide dorsiventrally, without scabrous margins6
6. Leaf lamina filiform; plant less than 20 cm tall; inflorescence up to 2 cm long*A*. *humilis*
6: Leaf lamina ensiform; plant 20–80 cm tall; inflorescence 2–10 cm long7
7. Inflorescence axis 0.7–1.2 mm wide; culms with up to 40 flowers; perianth in males 6–8.5 mm long, perianth in females 2–4.5 mm long*A. dioica* (=*A*. *gracilis* sp. 1).
7: Inflorescence axis 3–5 mm wide; culms with up to 200 flowers; perianth in males 3–3.5 mm long, perianth in females 2.2–2.5 mm long*A*. *laevis*

## Conclusions

Our study provides a remarkable example of locally common and conspicuous plants that were collected a long time ago, the collections being available in major public herbaria, but not properly analysed until the present study. The first collection of *A. dioica* was made nearly 200 years ago, but the clear difference between *A. dioica* and *A. gracilis* in the presence vs. absence of a conspicuous leaf ligule were not discovered until the present study.

The narrower species limits in the *A. gracilis* complex proposed in the present study provides a basis for future biogeographic analyses in *Anarthria*. Indeed, the ranges of *A. gracilis* s.str. and *A. grandiflora* are not overlapping and cover areas that differ in ecological conditions, including mean annual rainfall. In contrast, the range of *A. dioica* is wider, apparently disjunct and cannot be so readily explained. The range of *A. dioica* overlaps with the ranges of the other species. Clearly, historical factors should be taken into account along with climatic data in explaining species ranges. We believe that distribution patterns in *Anarthria* offer questions to be answered using the methods of phylogeography. We highlight the importance of collecting primary data in understanding diversity of even conspicuous and common plants.

The present study further highlights a need for the use of nuclear DNA markers in species-level phylogenetics (and phylogeography) of Restionaceae. We provide the first successful attempt in this field by use of the low-copy marker *at*103. The use of this nuclear marker allowed recognition of a clade comprising all ligulate species of *Anarthria* and evolutionary evaluation of the ligule character. Finally, we highlight a need for more detailed studies of morphology. For example, seed anatomy and surface morphology may provide additional features useful in the taxonomy of *Anarthria* to find potential morphological variation within *A*. *scabra*, *A*. *laevis* and *A*. *prolifera* since these species showed differences in nucleotide sequences according to our analysis. Studies on leaf morphology are also required in other groups of Restionaceae where the ligule character may be useful as well.

## Supplemental Information

10.7717/peerj.10935/supp-1 Supplemental Information 1 Material used in molecular and anatomical studies. GenBank accession numbers of sequences generated for this paper are in italics. Vouchers of specimens used for anatomical studies are in bold. All specimens are from Western Australia.

10.7717/peerj.10935/supp-2 Supplemental Information 2 Alignment of trnL-F sequences.

10.7717/peerj.10935/supp-3 Supplemental Information 3 Alignment of at103 sequences.

## Additional Information and Declarations

### Field Study Permissions

The following information was supplied relating to field study approvals (i.e., approving body and any reference numbers): Scientific collecting licenses and permissions to access reserve land were provided by the Department of Parks and Wildlife, Western Australia, now Department of Biodiversity, Conservation and Attractions (SW016575, SW017203, SW018172, CE006047).

### DNA Deposition

The following information was supplied regarding the deposition of DNA sequences: Alignments of DNA sequences of the two markers used in the article, including all newly produced sequences are available in the Supplemental Files. The *at*103 sequences described here are available at GenBank: MT775949 to MT775998. The *trn*L-F sequences described here are also available at GenBank: MT775887 to MT775948.

### Data Availability

The following information was supplied regarding data availability: The alignments of DNA sequences of the two markers used in the article, including all newly produced sequences are available in the Supplemental Files.
