# Supplementary material for: Two centuries from species discovery to diagnostic characters: molecular and morphological evidence for narrower species limits in the widespread SW Australian Anarthria gracilis complex (Restionaceae s.l./Anarthriaceae, Poales)
Source: PeerJ. 2021 Mar 8;9:e10935. doi: 10.7717/peerj.10935 (PMC7950204; doi:10.7717/peerj.10935)
Supplement: Supplemental Information 1 — GenBank accession numbers of sequences generated for this paper are in italics. Vouchers of specimens used for anatomical studies are in bold. All specimens are from Western Australia. [file peerj-09-10935-s001.doc]

**Appendix 1. Material used in molecular, anatomical and geographical studies.** The following format is used: taxon name, collector’s name and number (vouchers of specimens used for anatomical studies are indicated by asterisk), herbarium acronym and barcode, geographical location, GenBank accession numbers for *at*103 and *trn*L-F (–, –, when sample was not used in molecular analyses). Sequences generated for this paper are in italics. All specimens are from Western Australia.

***Anarthria dioica* (Steud.) C.I. Fomichev**

*A. dioica* (1), *Annels A.R. 1808* (PERTH 03176479), Pt 5059, Granite Road, 1.1 km E of Denmark River, 17 km NNW of Denmark, Map: Denmark 1:50,000, Ref: JO 135 17, –, –;

*A*. *dioica* (2), *Annels A.R. 3873* *ARA* (PERTH 05487064), Plot 5191, 200 m E, near junction of Frankland-Cranbrook Road and Yerriminup Road, –, –;

*A*. *dioica* (3), *Annels A.R. & Hearn R.W. 4723* *ARA* (PERTH 04128893), Little Lindsay, 800 m East of Stan's Road on "sand track", –, –;

*A*. *dioica* (4), *Annels A.R. & Macfarlane T.D. 5496* *ARA* (PERTH 04131029), 18 kms ENE of Rocky Gully on W edge of Reserve 26586, –, –;

*A*. *dioica* (5), *Ashby A.M. 3675* (PERTH 03311422), Stirling district. Stirling Range, –, –;

*A*. *dioica* (6), *Beauglehole A.C. ACB 49307* (PERTH 02198118), 23 km W of junction of Esperance-Norseman-Ravensthorpe Roads, ca 30 km NW of Esperance P.O. [Post Office], –, –;

*A*. *dioica* (7), *Blake S.T. 20861* (PERTH 02059312), Mount Barker, –, –;

*A*. *dioica* (8), *Briggs B.G. 508* (NSW 84040, PERTH 06326870), Stirling Range S of Chester Pass, 53 miles from (NNE of) Albany, –, –;

*A*. *dioica* (9), *Briggs B.G. 509* (NSW 84039, PERTH 06326889), Stirling Range S of Chester Pass, 53 miles from (NNE of) Albany, –, –;

*A*. *dioica* (10), *Briggs B.G. & Johnson L.A.S. BB 7653* (NSW, PERTH 02059428), 17 km NE of Manypeaks on South Coast Highway, –, –;

*A*. *dioica* (11), *Briggs B.G. & L. Johnson L.A.S. BB 7654* (NSW, PERTH 02059401), 17 km NE of Manypeaks on South Coast Highway, –, –;

*A*. *dioica* (12), *Briggs B.G. & L. Johnson L.A.S. BB 7854* (NSW, PERTH 02059681), Bremer Bay, NW side of township, –, –;

*A*. *dioica* (13), *Burgman M.A. & McNee S. MAB 1646* (PERTH 02059509), 20.5 km due SSE of Mount Burdett, –, –;

*A*. *dioica* (14), *Byrne G. 4816* (PERTH 08598371), Along Sandalwood Rd about 1.5 km from the Cape Riche camping area, –, *MT775911*;

*A*. *dioica* (15), *Byrne G. 4960* (PERTH 08774390), 9.6 km along Sandalwood Rd from the Cape Riche Camping Ground, *MT775963*, *MT775912*;

*A*. *dioica* (16), *Byrne G. 5531* (PERTH 08808112), Apex Lookout on Sukey Hill, Cranbrook, –, –;

*A*. *dioica* (17), *Byrne G. 5532* (PERTH 08808120), Apex Lookout on Sukey Hill, Cranbrook, –, –;

*A*. *dioica* (18), *Casson N. & Annels T. SC 51.14* (PERTH 04741358), 900 m E of Bussell Highway on Tanah Marah Rd and 200 m S, *MT775960*, *MT775908*;

*A*. *dioica* (19), *Cooper D. 202* (PERTH 05741815), SW Block, Ambergate Reserve, –, –;

*A*. *dioica* (20), *Crisp M.D. & Cook L.G. 10000 MDC* (PERTH 08084211), 2.8 km N along Forby S Road from Chester Pass Road, Stirling Range, Eyre district, –, –;

*A*. *dioica* (21), *Croxford E.J. 6450* (PERTH 04510399), Rest area W of Green Range Club, Hassell Highway, E of Albany, –, –;

*A*. *dioica* (22), *Croxford E.J. 7721 A* (PERTH 05738113), Attwell Park Reserve, Reddale Rd, 5 km NW of Albany, –, –;

*A*. *dioica* (23), *Davis R. 10441* (PERTH 06195946), Near junction of Warramurrup and Borden - Bremer Bay Road, –, –;

*A*. *dioica* (24), *Day C. & Casson N. W 158.17* (PERTH 04740661), Taylor Road, 400 m S of Muir Highway, –, –;

*A*. *dioica* (25), *Day C. & Casson N. W 158.20* (PERTH 04687515), Taylor Rd, 400 m S of Muir Highway, *MT775959*, *MT775907*;

*A. dioica* (26), *Drummond J. 344* (LE 0107695, four left hand plants), –, –;

*A*. *dioica* (27), *Fisher S.A. BNC 1414* (PERTH 08138761), Meelup, Dunsborough, –, –;

*A*. *dioica* (28), *Fomichev C.I. & Macfarlane T.D. WA408** (MW), 2 km N from Bakers Junction NR, *MT775964*, *MT775913*;

*A*. *dioica* (29), *Fomichev C.I. & Macfarlane T.D. WA654** (MW, PERTH), Ambergate, *MT775965*, *MT775914*;

*A*. *dioica* (30), *Fomichev C.I. & Macfarlane T.D. WA655** (MW), Ambergate, *MT775966*, *MT775915*;

*A*. *dioica* (31), *Fomichev C.I. & Macfarlane T.D. WA659** (MW), Ambergate, *MT775967*, *MT775916*;

*A*. *dioica* (32), *Fomichev C.I. & Macfarlane T.D. WA666** (MW), Ambergate, –, –;

*A*. *dioica* (33), *Fomichev C.I. & Macfarlane T.D. WA677** (MW), Ambergate, –, –;

*A*. *dioica* (34), *Gardner C.A. 273274275* (PERTH 02182920), Chillinup, –, –;

*A*. *dioica* (35), *Greuter* *23000* (PERTH 03214974), Fitzgerald River NP, E slopes of West Mount Barren, *MT775958*, –;

*A*. *dioica* (36), *Keighery G.J. 2599* (PERTH 02059460), Base of Toll Peak, Stirling Range, –, –;

*A*. *dioica* (37), *Keighery G.J. & Gibson N. 2258* (PERTH 05148960), 100 m S Wingebellup Road and Unicup Road intersection, Kululinup [Kulunilup] Nature Reserve, –, –;

*A*. *dioica* (38), *Keighery G.J. & Keighery B.J. 937* (PERTH 07708394), Korijekup CP, SE Harvey, *MT775961*, *MT775910*;

*A*. *dioica* (39), *Kenneally K.F. 6944* (PERTH 01190172), C. Milton's property, 3 km S of Mount Barker, 355 km S of Perth, –, –;

*A*. *dioica* (40), *McCallum Webster M. WA/362* (PERTH 04510372), By Hassell Highway, E of Albany, –, –;

*A*. *dioica* (41), *McCallum Webster M. WA/377* (PERTH 04510496), By Wellstead shop, Hassell Highway, E of Albany, –, –;

*A*. *dioica* (42), *Morrison A. s.n.* (PERTH 02059320), Conical Hill, Stirling Range, –, –;

*A*. *dioica* (43), *Morrison A. s.n.* (PERTH 02059576), Red Gum Pass, Stirling Range, –, –;

*A*. *dioica* (44), *Morrison A. s.n.* (PERTH 06241565), Between Tenterden and Solomon's Well, –, –;

*A*. *dioica* (45), *Newbey K.R. 4425* (PERTH 03132528), Northern boundary of Bremer Bay townsite, –, –;

*A*. *dioica* (46), *Phillips M.E. WA/62 1090* (PERTH 02059436), 90 km from Moora toward Jurien Bay, –, –;

*A. dioica* (47), *Preiss L.* *1803* (LE 01076945, 3^rd^, 4^th^ and 6^th^ plants from the left side of the sheet), “Albany” (Plantagenet), –, –;

*A. dioica* (48), *Preiss* *L. 1815* (LE 01076946, left plant), –, –;

*A*. *dioica* (49), *Rechinger K.H. 60320* (PERTH 02065428), Along Chester Pass Road, 70 km NNE Albany, –, –;

*A*. *dioica* (50), *Rechinger K.H. 60324* (PERTH 02065398), Along Chester Pass Road, 70 km NNE Albany, –, –;

*A*. *dioica* (51), *Royce R.D. 2910* (PERTH 02059614), Chapman Hill, Busselton district, –, –;

*A*. *dioica* (52), *Royce R.D. 3566* (PERTH 02059266), 3 miles N of Gibson, Esperance district, –, –;

*A*. *dioica* (53), *Sandiford E.M. EMS 426 A* (PERTH 05708389), Lateritic outcrop NE of Mt Eileen, SE corner of Mt Martin Regional Botanic Park, Albany, –, –;

*A*. *dioica* (54), *Sandiford E.M. EMS 426 B* (PERTH 05708435), Lateritic outcrop NE of Mt Eileen, SE corner of Mt Martin Regional Botanic Park, Albany, –, –;

*A*. *dioica* (55), *Stevens J. TM 101* (PERTH 07408684), 7 km N of Margaret River, 150 Tanah Marah Rd, south western end of property, –, *MT775909*;

*A*. *dioica* (56), *[d’]Urville J.D. s.n.* (P 00748634, 00748635, 00748636), Port du Roi Georges, N. Holl, –, –;

*A*. *dioica* (57), *Wardell-Johnson G. 2264 ARA* (PERTH 04295811), Near summit of granite outcrop, 19 km ESE of Mt Frankland, –, –;

*A*. *dioica* (58), *Wardell-Johnson G. 2264 ARA* (PERTH 04557166), Granite, near summit, 19 km ESE of Mt Frankland, –, –;

*A*. *dioica* (59), *Wilson P.G. 4326* (PERTH 02003716), 3 km W of Bremer Bay township, south coast, –, –;

*A*. *dioica* (60), *Wilson P.G. 4326a* (PERTH 02059606), 3 km W of Bremer Bay township, south coast, –, –;

***Anarthria gracilis* R.Br.**

*A*. *gracilis* s.str. (1), *Andrews C. s.n.* (PERTH 02059533), Torbay Junction [Albany], –, –;

*A*. *gracilis* s.str. (2), *Andrews C. s.n.* (PERTH 02059649, 02059711), Torbay Junction, between Albany and Denmark, –, –;

*A*. *gracilis* s.str. (3), *Andrews C. s.n.* (PERTH 06241573), Torbay Junction [Torbay Inlet], –, –;

*A*. *gracilis* s.str. (4), *Annels A.R. 184* (PERTH 02655330), Walpole Nornalup National Park, off Nut road, –, –;

*A*. *gracilis* s.str. (5), *Annels A.R. 593 ARA* (PERTH 02655349, 02655365), Track off Twin Creek Road, 13 km E of Walpole, Plot 1017, Walpole-Nornalup National Park, –, –;

*A*. *gracilis* s.str. (6), *Annels A.R. 1413 ARA* (PERTH 04765842), Plot 4229, on Mountain Road, –, –;

*A*. *gracilis* s.str. (7), *Beauglehole A.C. ACB 12759* (PERTH 02335085), Albany, Gull Rock Road 4.8 km from main Road, –, –;

*A*. *gracilis* s.str. (8), *Blake S.T. 20691* (PERTH 02059339), Cannington, –, –;

*A*. *gracilis* s.str. (9), *Bright D. & Day C. SC 197.18* (PERTH 04723287), S side of Scott River Road, 1.4 km W of 90 degree bend in road, –, –;

*A*. *gracilis* s.str. (10), *Briggs B.G. 9588* (NSW 608395, PERTH 07313039), Muirs Highway c. 96 km WNW of Mount Barker, –, –;

*A*. *gracilis* s.str. (11), *Briggs* *B.G. 9958* (NSW 784576, PERTH 08506531), Woogenellup Rd E of Mt Barker, 0.9 km SW of Duck Rd, *MT775978*, *MT775926*;

*A*. *gracilis* s.str. (12), *Brown R. (Bennett 5841)* (BM 000991239, 000991240, E 00346011, K 001056262, 001056263, MEL 14501), King Georges Sound, –, –;

*A*. *gracilis* s.str. (13), *Byrne* *B.G.* *4137* (PERTH 08454507), Lower King Rd between Hooper Rd and Mercer Rd, *MT775976*, –;

*A*. *gracilis* s.str. (14), *Cashmore R. 19* (PERTH 02065339), Near Nornalup, –, –;

*A*. *gracilis* s.str. (15), *Casson* *N. & Annels* *A.R.* *SC 40.5* (PERTH 04722558), 1.6 km S along Caves Rd from Juniper Rd and 150 m W along track through swamp, *MT775974*, –;

*A*. *gracilis* s.str. (16), *Casson N. & Godden C. P 66.2* (PERTH 04735374), 50 m S of Deeside Coast Road and Dog Roads on Deeside Coast Road, –, –;

*A*. *gracilis* s.str. (17), *Casson N. & Kershaw K. W 169.14* (PERTH 04699076), 1.2 km W of junction of Chokerup Siding Road and Chokerup Road, Walpole Region, –, –;

*A*. *gracilis* s.str. (18), *Churchill D. s.n.* (PERTH 02059630), Boggy Lake [9 km SW of Walpole, 600 m NW of Mount Hopkins and 8 km SE Crystal Springs. R.W. Hearn, –, –;

*A*. *gracilis* s.str. (19), *Cranfield R.J. 10371* (PERTH 04439082), 9 km SSW of Mount Johnston, –, –;

*A*. *gracilis* s.str. (20), *Cranfield* *R.J. & Ward B.G. 24872* (PERTH 08475954), Nornalup Rd, Trent, *MT775977*, *MT775925*;

*A*. *gracilis* s.str. (21), *Cranfield* *R.J. & Ward B.G. 25044* (PERTH 08508119), Plot WFM 03, London forest block, 2 km S of Mountain Road along Renzo Road extension, –, –;

*A*. *gracilis* s.str. (22), *Cranfield* *R.J. & Ward B.G. 24872* (PERTH 08475954), Nornalup road, Trent, –, –;

*A*. *gracilis* s.str. (23), *Cranfield* *R.J. & Ward B.G. WFM 51* (PERTH 07102372), Plot 3, London forest block, 2 km S of Mountain Road along Renzo Road extension, –, –;

*A*. *gracilis* s.str. (24), *Cranfield* *R.J. & Ward B.G. WFM 189* (PERTH 07099762), Plot 6, London forest block, 500 km W of Nornalup Road along gravel pit road, –, –;

*A*. *gracilis* s.str. (25), *Cranfield* *R.J. & Ward B.G. WFM 190* (PERTH 07099770), Plot 6, London forest block, 500 km W of Nornalup Rd along gravel pit road, *MT775975*, –;

*A*. *gracilis* s.str. (26), *Crisp M.D. 5328* (PERTH 02059517), 17 km WSW of Walpole, 4 km N of Point Irwin, –, –;

*A*. *gracilis* s.str. (27), *Crisp M.D. 5329* (PERTH 02059657), 17 km WSW of Walpole, 4 km N of Point Irwin, Warren district, –, –;

*A*. *gracilis* s.str. (28), *Croxford E.J. 1106 A* (PERTH 04486617), Keith Road, Hay River, Shire of Denmark, –, –;

*A*. *gracilis* s.str. (29), *Croxford E.J. 7306* (PERTH 05479878), Cemetery Reserve, Allambie Park, Lower King Road, Albany, –, –;

*A*. *gracilis* s.str. (30), *Croxford E.J. 8243* (PERTH 05839181), Abandoned Sewage Farm, Collingwood Road, 7 km NE of Albany, –, –;

*A*. *gracilis* s.str. (31), *Davis* *R. 171* (PERTH 04396782), 12 km NNE of Augusta, *MT775973*, –;

*A*. *gracilis* s.str. (32), *Davis R. RD 1849* (PERTH 04590562), Down Dump road, from Wheatley Coast road, 5 km E of Quininup, –, –;

*A*. *gracilis* s.str. (33), *Ellery P. & Godden C. W 5.10* (PERTH 04677013), 50 m S of Ficifolia Road, 1.2 km W of its junction with Peaceful Bay Road, Walpole Region, –, –;

*A*. *gracilis* s.str. (34), *Fomichev C.I. & Macfarlane T.D. WA413* (MW), Gull Ruck National Park, Mount Richard Rd, c. 1.4 km W of its junction with Nanarup Rd, *MT775980*, *MT775928*;

*A*. *gracilis* s.str. (35), *Fomichev C.I. & Macfarlane T.D. WA414** (MW), Gull Ruck National Park, Mount Richard Rd, c. 1.4 km W of its junction with Nanarup Rd, *MT775981*, *MT775929*;

*A*. *gracilis* s.str. (36), *Fomichev C.I. & Macfarlane T.D. WA415* (MW), Gull Ruck National Park, Mount Richard Rd, c. 1.4 km W of its junction with Nanarup Rd, *MT775982*, *MT775930*;

*A*. *gracilis* s.str. (37), *Fomichev C.I. & Macfarlane T.D. WA690** (MW), South Western Highway, c. 4.5 km NE of its junction with Middleton Rd, *MT775983*, *MT775931*;

*A*. *gracilis* s.str. (38), *Fomichev C.I. & Macfarlane T.D. WA691* (MW), South Western Highway, c. 4.5 km NE of its junction with Middleton Rd, *MT775984*, *MT775932*;

*A*. *gracilis* s.str. (39), *Fomichev C.I. & Macfarlane T.D. WA692* (MW), South Western Highway, c. 4.5 km NE of its junction with Middleton Rd, *MT775985*, *MT775933*;

*A*. *gracilis* s.str. (40), *Fomichev C.I. & Macfarlane T.D. WA700** (MW), Shannon Airstrip, *MT775986*, *MT775934*;

*A*. *gracilis* s.str. (41), *Fomichev C.I. & Macfarlane T.D. WA701** (MW), Shannon Airstrip, *MT775987*, *MT775935*;

*A*. *gracilis* s.str. (42), *Fomichev C.I. & Macfarlane T.D. WA702* (MW), Shannon Airstrip, *MT775988*, *MT775936*;

*A*. *gracilis* s.str. (43), *Fomichev C.I. & Macfarlane T.D. WA707** (MW), Shannon Airstrip, –, –;

*A*. *gracilis* s.str. (44), *Fomichev C.I. & Macfarlane T.D. WA715** (MW), Shannon Airstrip, –, –;

*A*. *gracilis* s.str. (45), *Fomichev C.I. & Macfarlane T.D. WA728* (MW), South Western Highway and Beardmore Rd, *MT775989*, *MT775937*;

*A*. *gracilis* s.str. (46), *Fomichev C.I. & Macfarlane T.D. WA729* (MW), South Western Highway and Beardmore Rd, *MT775990*, *MT775938*;

*A*. *gracilis* s.str. (47), *Fomichev C.I. & Macfarlane T.D. WA730** (MW), South Western Highway and Beardmore Rd, *MT775991*, *MT775939*;

*A*. *gracilis* s.str. (48), *Gibson N. & Lyons M. 1141* (PERTH 03132854), Northern boundary firebreak of Gingilup Swamps Nature Reserve, 3.5 km SE of the NW corner of the reserve (Plot: gsnr7), –, –;

*A*. *gracilis* s.str. (49), *Godden M. & Casson N. W 106.7* (PERTH 04706153), Denmark – Mount Barker Road, 200 m from Blue Lake Road intersection, Plot is 60 m W of road, –, –;

*A*. *gracilis* s.str. (50), *Hoyle N. 1179* (PERTH 01953419), 25 km W along Stirling Range Drive from intersection with Chester Pass Road, –, –;

*A*. *gracilis* s.str. (51), *Jackson S.W. s.n.* (PERTH 02182947), Bow River, South West, –, –;

*A*. *gracilis* s.str. (52), *Jurjevich P.A. 701* (PERTH 04849744), Site 99, 8 km ENE of Augusta, –, –;

*A*. *gracilis* s.str. (53), *Keighery G.J. 10854* (PERTH 04120493), Yelverton Forest; Blythe Road, 25 km NW Margaret River, –, –;

*A*. *gracilis* s.str. (54), *Kenneally K.F. 1117* (PERTH 01190261), 226 miles S of Perth off Albany Highway, ca. 4 miles S of Mount Barker townsite, C. Milton's property, –, –;

*A*. *gracilis* s.str. (55), *Kenneally K.F. 2374* (PERTH 01190687), C. Milton's property, 3 km S of Mount Barker, 355 km S of Perth, –, –;

*A*. *gracilis* s.str. (56), *Koch M. 2625* (PERTH 02138026, 02138077), Pemberton, –, –;

*A*. *gracilis* s.str. (57), *Loneragan W.A. 59* (PERTH 06696414), Mersea Lake, ca 12 miles S of Bridgetown near Wilgarrup, –, –;

*A*. *gracilis* s.str. (58), *Macfarlane T.D. & Fuhrer B. TDM 3047* (PERTH 04949153), Muir Highway, beside Lake Muir, 3.9 km E of Thompson Road, –, –;

*A*. *gracilis* s.str. (59), *McCallum Webster M. WA/216* (PERTH 02182963), Near Perkins road turning on the Lower Albany/Denmark road, Warren, –, –;

*A. gracilis* s.str. (60), *Preiss L. 1803* (LD 1354037, LE 01076952), prope oppidulum Albany, Plantagenet, –, –;

*A. gracilis* s.str. (61), *Preiss L.* *1803 & 1815* (LE 01076945, 1^st^, 2^nd^, 5^th^ and 7^th^ plants from the left side of the sheet), “Albany” (Plantagenet), –, –;

*A. gracilis* s.str. (62), *Preiss* *L. 1815* (LD 1746884, LE 01076946, right plant), prope urbeculum Albany, Plantagenet, –, –;

*A. gracilis* s.str. (63), *Preiss L. 1816* (LE 01076947), ad Stirling’s terrace, Plantagenet, –, –;

*A*. *gracilis* s.str. (64), *Pullen R. 9902* (PERTH 04057643), Scott River plains, E of Augusta, –, –;

*A*. *gracilis* s.str. (65), *Redwood K.A. 288* (PERTH 04788176), Site 146, W off Denbarker Road, –, –;

*A*. *gracilis* s.str. (66), *Redwood K.A. 469* (PERTH 04795865), Site 130, S of Beardmore Road, E of South Western Highway, –, –;

*A*. *gracilis* s.str. (67), *Royce R.D. 2386* (PERTH 06241549, 06241557), Palgarup, –, –;

*A*. *gracilis* s.str. (68), *Royce R.D. 2941* (PERTH 02059541), Scott River Plains, –, –;

*A*. *gracilis* s.str. (69), *Royce R.D. 2942* (PERTH 06326862), Plain between the Blackwood and Scott Rivers, –, –;

*A*. *gracilis* s.str. (70), *Smith G.T. & Moore L.A. s.n.* (PERTH 05319536, 05319579), Section I, along the western boundary of Two Peoples Bay Nature Reserve, –, –;

*A*. *gracilis* s.str. (71), *Smith G.T. & Moore L.A. s.n.* (PERTH 05319587), Section J, along the western boundary of Two Peoples Bay Nature Reserve, –, –;

*A*. *gracilis* s.str. (72), *Smith G.T. & Moore L.A. s.n.* (PERTH 06230121), Western boundary, Section J, Two Peoples Bay Nature Reserve, –, –;

*A*. *gracilis* s.str. (73), *Sokoloff D.D. & Macfarlane T.D. WA19* (MW 0940601), between Augusta and Albany, South Western Highway and Beardmore Rd intersection, *MT775979*, *MT775927*;

*A*. *gracilis* s.str. (74), *Wardell-Johnson G. GWJ 119* (PERTH 05471494), Plot 5455, W of Betty's Beach on Two Peoples Bay, Boulder Hill, –, –;

*A*. *gracilis* s.str. (75), *Wardell-Johnson G. & Annels A.R. 2559 ARA* (PERTH 04437330), Plot 5454 Boulder Hill, –, –;

*A*. *gracilis* s.str. (76), *Webb A. AW 2268* (PERTH 07488726), Margaret River Flats, on Adelaide Road, ca 1.8 km North of junction with Margaret Road, –, –;

***Anarthria grandiflora* Nees**

*A. grandiflora* (1) *Briggs B.G. 9603* (NSW 716626, PERTH 07312733), Woolka Rd 3 km WSW of junction with Cooljarloo Rd, NW of Cataby, *MT775992*, *MT775941*;

*A. grandiflora* (2), *Briggs B.G. 9939* (NSW 784465, PERTH 08083304), Bibby Road near Drummonds Reserve, –, –;

*A. grandiflora* (3), *Briggs B.G. & Johnson L.A.S. BB 7626* (NSW 7901722, PERTH 02059525), Ficifolia Road, ca 8 km SE of Nornalup, –, –;

*A. grandiflora* (4), *Carlquist S. 5629* (PERTH 02059304, 02059282), Along Brook road, near junction with Grove Road [Kenwick], –, –;

*A. grandiflora* (5), *Drummond J. s.n.* (B 100278880, CGE 05073, K 001056264), Swan River [Colony], –, –;

*A. grandiflora* (6), *Drummond J. 904* (LE 01076951, the extreme right plant only), –, –;

*A. grandiflora* (7), *Fitzgerald W.V. s.n.* (NSW 60725, PERTH 02059371), Canning Plains [Perth], –, –;

*A. grandiflora* (8), *Fomichev C.I. & Macfarlane T.D. WA642** (MW), Bibby Rd [SE of Badgingarra], –, *MT775942*;

*A. grandiflora* (9), *Fomichev C.I. & Macfarlane T.D. WA643** (MW), Bibby Rd [SE of Badgingarra], –, –;

*A. grandiflora* (10), *Fomichev C.I. & Macfarlane T.D. WA644** (MW), Bibby Rd [SE of Badgingarra], *MT775994*, *MT775944*;

*A. grandiflora* (11), *Fomichev C.I. & Macfarlane T.D. WA646* (MW, PERTH), intersection of Brand Highway and Cataby Rd, *MT775995*, *MT775945*;

*A. grandiflora* (12), *Fomichev C.I. & Macfarlane T.D. WA647** (MW), intersection of Brand Highway and Cataby Rd, *MT775996*, *MT775946*;

*A. grandiflora* (13), *Fomichev C.I. & Macfarlane T.D. WA648** (MW), intersection of Brand Highway and Cataby R, *MT775997*, *MT775947*;

*A. grandiflora* (14), *Griffin E.A. 8354* (PERTH 03510905), Cadda Rd, E of Munbinea Rd, W of Badgingarra, –, *MT775940*;

*A. grandiflora* (15), *Johnson L.A.S. 8139* (NSW, PERTH 02059568), N of Cataby Roadhouse in Badgingarra area, –, –;

*A. grandiflora* (16), *Keighery G.J. 2534* (PERTH 02059444), Cervantes to Jurien Bay road, 5 km N of Hill River Crossing, –, –;

*A. grandiflora* (17), *MacPherson G.J. LE 47.1* (PERTH 06380603), Site 47, 6 km E of Tombstone rocks, –, –;

*A. grandiflora* (18), *Rechinger K.H. 59515* (PERTH 02065401), Kenwick, Nature Reserve owned by Botany Department, UWA, –, –;

*A. grandiflora* (19), *Spjut R. & Smith R. 12447* (PERTH 06375286), E of Park boundary, ca 11 km W of Brand Highway along Wongonderrah Road 1.5 km W from junction with N road to Stewart. 1-2 km SW along sand track, Nambung National Park, –, –;

***Anarthria* *humilis* Nees**

*A. humilis* (1), *Annels A.R. ARA 1458* (PERTH 04568079), Plot 4027, [Break Road ca 2.6 km NE of Nornalup Road, NE of Walpole], –, –;

*A. humilis* (2), *Aplin T.E.H. et al. 3252* (PERTH 02059762), Fitzgerald River, ca 70 miles (112.7 km) ESE of Ongerup, –, –;

*A. humilis* (3), *Beauglehole A.C. ACB 49238* (PERTH 02065363), 39 km SW of Fitzgerald, Ravensthorpe-Ongerup Road, –, –;

*A. humilis* (4), *Beauglehole A.C. ACB 49193* (PERTH 02065371), 39 km SW of Fitzgerald, Ravensthorpe-Ongerup Road, –, –;

*A. humilis* (5), *Bennett M. 1012* (PERTH 07333854), c. 80 m N of firebreak on track between firebreak and Hammersley Drive Hopetoun, –, –;

*A. humilis* (6), *Bowler H. 651* (PERTH 05770467), Ellis Brook Valley Reserve, –, –;

*A. humilis* (7), *Briggs B.G. 387* (PERTH 02059932), Lucky Bay, E of Esperance, –, –;

*A. humilis* (8), *Briggs B.G. 502* (PERTH 02059959), Chester Pass, Stirling Range, Bluff Knoll turnoff from Albany-Borden road, –, –;

*A. humilis* (9), *Briggs B.G. 6315* (PERTH 02060043), Badgingarra National Park, 6.5 km S of New Badgingarra, –, –;

*A. humilis* (10), *Briggs B.G. 6343* (PERTH 02060035), 4.5 km N of Cockleshell Gully, W of Mount Peron (NE of Jurien), –, –;

*A. humilis* (11), *Briggs B.G. 6355* (PERTH 02060027), 7 km W of Mount Lesueur, 5.5 km along track from Cockleshell Gully to main Jurien road, –, –;

*A. humilis* (12), *Briggs 9476* (NSW 437356, PERTH 08506361), Brand Highway just S of Tootbardie Rd, *MT775962*, GQ409004;

*A. humilis* (13), *Briggs B.G. & Johnson L.A.S. BB 7472* (PERTH 03119807), Ca 2 km SW of Mount Lesueur, 5 km E of Cockleshell Gully road on track towards Mount Lesueur, –, –;

*A. humilis* (14), *Briggs B.G. & Johnson L.A.S. BB 7646* (PERTH 02060019) 2.7 km SW of Manypeaks on South Coast Highway, 0.7 km SW of Bettys Beach turn, –, –;

*A. humilis* (15), *Briggs B.G. & Johnson L.A.S. BB 7671* (PERTH 02059975), 38 km ENE of Jerramungup, –, –;

*A. humilis* (16), *Briggs B.G. & Johnson L.A.S. BB 7821* (PERTH 02059967), S of Lake Chidnup, 27.4 km N of Highway 1, on road to Lake King, –, –;

*A. humilis* (17), *Briggs B.G. & Johnson L.A.S. BB 7909* (PERTH 02059991), Gnowellen Road, 14.5 km NW of Chillinup Road junction, due E of Ellen Peak (Stirling Range), –, –;

*A. humilis* (18)*, Briggs B.G. & Johnson L.A.S. BGB 7822* (PERTH 03555399), South of Lake Chidnup, 27.4 km N of Highway 1 on road to Lake King, –, –;

*A. humilis* (19), *Burgman M.A. & McNee S. MAB 2119* (PERTH 02060108), 34.5 km due ESE of Muckinwobert Rock, 16.5 km S of West Point road on Melaleuca Road, –, –;

*A. humilis* (20), *Byrne G. 4772* (PERTH 08599459), Gravel pit on Chillinup Road, NW of Old Boundary Road, –, –;

*A. humilis* (21), *Byrne G. 4775* (PERTH 08602557), Gravel pit on Chillinup Road, NW of Old Boundary Road, –, –;

*A. humilis* (22), *Byrne G. 5585* (PERTH 08810486), Corackerup Road, Corackerup Reserve, –, –;

*A. humilis* (23), *Craig G.F. 6338* (PERTH 07540825), ca 15.5 km S of South Coast Highway on Hopetoun - Ravensthorpe Road, E road reserve, –, –;

*A. humilis* (24), *Cranfield R.J. 2277* (PERTH 02059274), H.T. power lines SE of Gosnells Quarries, –, –;

*A. humilis* (25), *Cranfield R.J. & Spencer P.J. 8288* (PERTH 02474263), 2 km SW of Jurien Bay turnoff, 3.7 km W of Brand Highway along track, –, –;

*A. humilis* (26), *Crisp M.D. 5234* (PERTH 02059878), 4 km SE of Porongurup, Stirling district, –, –;

*A. humilis* (27), *Crisp M.D. 5235* (PERTH 02059797), 4 km SE of Porongurup, Stirling district, –, –;

*A. humilis* (28), *Crowley V. DKN 26* (PERTH 04744659), Reserve, E side, corner Piesseville - Tarwonga Road and Narrogin Road, NNE of Arthur River, –, –;

*A. humilis* (29), *Croxford E.J. 4159* (PERTH 04510402), Dongolocking Road reserve, Shire of Dumbleyung, –, –;

*A. humilis* (30), *Eichler Hj. 19826* (PERTH 02059908), Near Howick Hill, 5.5 km E of the woolsheds of Mount Howick Station, Location 259 (ca 100 km E of Esperance), –, –;

*A. humilis* (31), *Eichler Hj. 19827* (PERTH 02059940), Near Howick Hill, 5.5 km E of the woolsheds of Mount Howick Station, Location 259 (ca 100 km E of Esperance), –, –;

*A. humilis* (32), *Elkington J. C 28* (PERTH 04167198), 18 km N of Cataby, –, –;

*A. humilis* (33), *Fomichev C.I. & Macfarlane T.D. WA407* (MW), Stirling Range, Bluff Knoll, 500 m from Chester Pass, –, *MT775917*;

*A. humilis* (34), *Fomichev C.I. & Macfarlane T.D. WA605* (MW), Ryansbrook, 3 km W of Douglas Rd and Shamrock Rd intersection, *MT775968*, *MT775918*;

*A. humilis* (35), *Fomichev C.I. & Macfarlane T.D. WA606* (MW), 30 km N of Kojonup, 3 km W of Douglas Rd and Shamrock Rd intersection, *MT775969*, *MT775919*;

*A. humilis* (36), *Fomichev C.I. & Macfarlane T.D. WA607* (MW), 30 km N of Kojonup, 3 km W of Douglas Rd and Shamrock Rd intersection, *MT775970*, *MT775920*;

*A. humilis* (37), *Fomichev C.I. & Macfarlane T.D. WA608* (MW), 30 km N of Kojonup, 3 km W of Douglas Rd and Shamrock Rd intersection, –, *MT775921*;

*A. humilis* (38), *Fomichev C.I. et al. WA589* (MW 0940608), S of Jurien Rd and E of Black Arrow Rd, –, *MT775922*;

*A. humilis* (39), *George A.S. 6366* (PERTH 02059487), Paper Collar Gully, Chester Pass, Stirling Range, –, –;

*A. humilis* (40), *George A.S. 14604* (PERTH 02060116), Ca 6 km W of Mount Lesueur, –, –;

*A. humilis* (41), *Griffin E.A. 1918* (PERTH 02060078), SE slope of Mount Lesueur, NE of Jurien, –, –;

*A. humilis* (42), *Griffin E.A. 2002* (PERTH 02060124), Hill, 1 km NW of Mount Lesueur, NE of Jurien, –, –;

*A. humilis* (43), *Griffin E.A. 4898* (PERTH 01193597), Mullering Road, NW of Dandaragan, AMG 50 JLM630151 (Dandaragan 1:50,000 sheet), –, –;

*A. humilis* (44), *Hislop M. 1718 A* (PERTH 05563569), Hi Vallee property (D. & J. Williams) Warradarge, southern end of main valley, –, –;

*A. humilis* (45), *Hislop M. 1718* *B* (PERTH 05563577), Hi Vallee property (D. & J. Williams) Warradarge, southern end of main valley, –, –;

*A. humilis* (46), *Hnatiuk R.J. 760027* (PERTH 02060132), 1 km W of Brand Highway on Mimegarra Road, –, –;

*A. humilis* (47), *Hnatiuk R.J. 760958* (PERTH 02059843), Merivale Road [E of Esperance], –, –;

*A. humilis* (48), *Hnatiuk R.J. 761117* (PERTH 02060000), 11 km N of Point Malcolm, –, –;

*A. humilis* (49), *Hort F. & Hort J. 3199* (PERTH 08361347), Gravel Reserve, Brand Highway, Cataby, Shire of Dandaragan; the E side of the highway ca 3 km S of Yandin Road, –, –;

*A. humilis* (50), *Hort F. & Hort J. 3200* (PERTH 08361355), Gravel Reserve, Brand Highway, Cataby, Shire of Dandaragan: the E side of the highway ca 3 km S of Yandin Road, –, –;

*A. humilis* (51), *Hort F. & Hort J. 3647* (PERTH 08395039), Monadnocks CON, Pike Road, Wandering; 3.5 km E of Watershed Road then trek 500 m N to the base of large granite outcrop, –, –;

*A. humilis* (52), *Jackson E.N.S. 1272* (PERTH 02059886), Buyi Billanak Homestead. Buyi Billanak Homestead is ca 12 km SE of Condingup Peak (Condingup Peak is ca 65 km E of Esperance), –, –;

*A. humilis* (53), *Johnson L.A.S. 8143* (PERTH 02060086), N of Regans Ford, towards Cataby Roadhouse, –, –;

*A. humilis* (54), *Johnson L.A.S. 8144* (PERTH 02060051), N of Regans Ford towards Cataby Roadhouse, –, –;

*A. humilis* (55), *Keighery B.J. 119 B* (PERTH 03119793), Brand Highway on eastern side road approximately 5 km S Mimegarra Road AMG 50JLL647925 Dandaragan 1:100,000, –, –;

*A. humilis* (56), *Keighery B.J. & Keighery G.J. 12* (PERTH 04497791), Remnant bushland Lambert Lane, Wungong, near railway line, S Armadale (adj. to plot lamb1), –, –;

*A. humilis* (57), *Keighery G.J. 1170* (PERTH 02059924), Salt River Road, opposite Peak Donnelly, Stirling Ranges, –, –;

*A. humilis* (58), *Keighery G.J. 7046* (PERTH 02065320), 5 km E of Byford on Nettleton Road, –, –;

*A. humilis* (59), *Keighery G.J. 11405* (PERTH 01196014), Cheyne Beach area, Bluff Point Road, –, –;

*A. humilis* (60), *Keighery G.J. & Alford J.J. 1802* (PERTH 01414577), Sukey's Peak, 5 km E of Cranbrook, –, –;

*A. humilis* (61), *Keighery G.J. & Gibson N. 5272* (PERTH 06883869), On N side of powerline, 75 m E of junction of Neds-Corner and Yerritup Roads, Reserve 31766, c. 14 km NW of Stokes Inlet. [Plot - GP13], –, –;

*A. humilis* (62), *Keighery G.J. & Gibson N. 5274* (PERTH 06883885), On N side of South-Coast Highway, 2.4 km W of Mills Road. Munglinup Nature Reserve, c. 8 km WNW of Munglinup. [Plot - GP18], –, –;

*A. humilis* (63), *Keighery G.J. & Gibson N. 5273* (PERTH 06883877), On W side of Fox Road, 2.6 km S of Brockway Road. Helms Arboretum, ca 16 km NNW of Esperance. [Plot - ES01], –, –;

*A. humilis* (64), *Markey A. & Bayliss B. NIB 9631* (PERTH 08994986), 1.5 km NNW of Hellfire Bay carpark, 1.9 km E of Mt Le Grand summit, 5.9 km W of Lucky Bay campsite, Cape Le Grand National Park, 29 km SE of Esperance township, Esperance Plains IBRA bioregion, –, –;

*A. humilis* (65), *Nelson E.C. ANU 16565* (PERTH 02060582), Mississippi Bay, Cape Le Grande National Park, Esperance, –, –;

*A. humilis* (66), *Nelson E.C. ANU 16614* (PERTH 02060574), Base of Frenchman Peak, Cape Le Grande National Park, –, –;

*A. humilis* (67), *Newbey K.R. 3762* (PERTH 04545478), 1.5 miles SE of Mount Maxwell, Fitzgerald River National Park, –, –;

*A. humilis* (68), *Newbey K.R. 3820* (PERTH 04545443), 5 Km SE of Bivouac Rocks, Fitzgerald River National Park, –, –;

*A. humilis* (69), *Newbey K.R. 4120* (PERTH 03091643), 22 km E of Gnowangerup, –, –;

*A. humilis* (70), *Newbey K.R. 4262* (PERTH 02059479), N boundary of Bremer Bay townsite, –, –;

*A. humilis* (71), *Nunn L.J. 208* (PERTH 01953257), Kau Rock Road Reserve, 0.3 km along Kau Rock Road from Coolinup Road, –, –;

*A. humilis* (72), *Orchard A.E. 1591* (PERTH 02059894), Near western border of Shire of Esperance, Eucla division, –, –;

*A. humilis* (73), *Paust S. 715* (PERTH 02059827), 40 miles W of Ravensthorpe, –, –;

*A. humilis* (74), *Royce R.D. 3566* (PERTH 02059770), 3 miles N of Gibson, Esperance district, –, –;

*A. humilis* (75), *Royce R.D. 8685* (PERTH 02059916), Cape Le Grand National Park, E of Esperance, –, –;

*A. humilis* (76), *Royce R.D. 8706* (PERTH 01953265), Cape Le Grand National Park, E of Esperance, –, –;

*A. humilis* (77), *Seabrook J. 608* (PERTH 02059983), Helena Valley, upper gorge, –, –;

*A. humilis* (78), *Solomon I. 614* (PERTH 02995395), Near gravel pit above Lucky Bay, –, –;

*A. humilis* (79), *Tauss C. 28* (PERTH 05948606), Near track from Cape Le Grand Rangers house to windmill - E of Rangers Station, Cape Le Grand National Park, –, –;

*A. humilis* (80), *Turley C.D. 7/899* (PERTH 05493099), Helms Arboretum, 17 km N of Esperance, –, –;

*A. humilis* (81), *Turley C.D. & Hoggart R.M. 23/504-10* (PERTH 08258384), Fox Road, Helms Arboretum, 17 km N of Esperance, –, –;

*A. humilis* (82), *Wajon J.E. 2459* (PERTH 08962774), Private property, Boxwood Hill-Ongerup Road, 200 m off middle track on track to white dam, 200 m W of paddock, –, –;

*A. humilis* (83), *Warren G. & Rose P. 754* (PERTH 06937233), Shire sand pit on N side of Contine Road (Reserve 20020), Narrogin, –, –;

*A. humilis* (84), *Whibley D.J.E. 5277* (PERTH 02060094), Ca 20 km W of Ongerup, Stirling district, –, –;

*A. humilis* (85), *Williams A. 22* (PERTH 05137144), Jerdacuttup town, W side of cemetery, –, –;

*A. humilis* (86), *Williams A. 148* (PERTH 05455928), 52 km SE of Ravensthorpe, ca 2 km W of Jerdacuttup School and Wheat Bin, –, –;

*A. humilis* (87), *Williams A. 283* (PERTH 05794617), Gravel pit, Jerdacuttup Road, near Woodstock Shed, ca 40 km SE of Ravensthorpe, –, –;

*A. humilis* (88), *Wilson P.G. 4388d* (PERTH 02059851), Along No. 2 Rabbit Fence, ca 40 km SSE of Jerramungup-Ravensthorpe road and 30 km N of Bremer Bay, –, –;

*A. humilis* (89), *Wilson P.G. 5413* (PERTH 02059789), 62 km W of Ravensthorpe on Ongerup road, –, –;

*A. humilis* (90), *Wilson P.G. 5414* (PERTH 02059754), 62 km W of Ravensthorpe on Ongerup road, –, –;

*A. humilis* (91), *Wilson P.G. 5502* (PERTH 02059819), 3 km N of Hopetoun, –, –;

*A. humilis* (92), *Wilson P.G. 6927* (PERTH 02059800), 63 km SE of Lake King township on Ravensthorpe road, –, –;

*A. humilis* (93), *Wilson P.G. 6928* (PERTH 02059746), 63 km SE of Lake King township on Ravensthorpe road, –, –;

*A. humilis* (94), *Wilson P.G. 7841* (PERTH 02059835), 80 km W of Esperance on Esperance to Ravensthorpe road, –, –;

*A. humilis* (95), *Wilson P.G. 7842* (PERTH 02059738), 80 km W of Esperance on Esperance-Ravensthorpe road, –, –;

***Anarthria humilis* (?×*A. dioica*)**

*A. humilis* (?×*A. dioica*) (1), *Briggs B.G. 7471* (NSW 406625, PERTH 05962145), 5 km E of Cockleshell Gully Road on track towards Mount Lesueur, c. 2 km SW of Mount Lesueur, –, –;

*A. humilis* (?×*A. dioica*) (2), *Briggs B.G. 9940B* (NSW 784467, PERTH 08287899), NW foot of Mount Lesueur, Cockleshell Gully Rd off Scenic Drive, Mount Lesueur NP, *MT775971*, *MT775923*;

*A. humilis* (?×*A. dioica*) (3), *Fomichev C.I. & Macfarlane T.D. WA439** (MW), Cockleshell Gully Rd, *MT775972*, *MT775924*;

***Anarthria* *laevis* R.Br.**

*A. laevis* (1), *Briggs G.B. 9843* (NSW), Chillinup Rd between Gnowellen Rd and Kojaneerup Spring Rd, –, KF218197;

*A. laevis* (2), *Fomichev C.I. & Macfarlane T.D. WA641* (MW), Bibby Rd [SE of Badgingarra], *MT775957*, *MT775906*;

*A. laevis* (3), *Wheeler M. et al. MW 308* (PERTH 07963009), Wandoo NP survey site 4, quadrat 1, –, *MT775905*;

***Anarthria* *polyphylla* Nees**

*A. polyphylla* (1), *Briggs B.G. 446* (PERTH 02060590), 10 miles SW of Ravensthorpe on Ongerup road, –, –;

*A. polyphylla* (2), *Briggs B.G. 448* (PERTH 02060639), 10 miles SW of Ravensthorpe on Ongerup road, –, –;

*A. polyphylla* (3), *Briggs B.G. 450* (PERTH 04207785), 10 miles (16 km) SW of Ravensthorpe on Ongerup road, –, –;

*A. polyphylla* (4), *Briggs B.G. 488* (PERTH 01742124), Ca 20 miles SE of Ongerup, –, –;

*A. polyphylla* (5), *Briggs B.G. 489* (PERTH 02060701), Ca 20 miles SE of Ongerup, –, –;

*A. polyphylla* (6), *Briggs B.G. 495* (PERTH 02060671), 11 miles SE of Gnowangerup on Borden road, –, –;

*A. polyphylla* (7), *Briggs B.G. 503* (PERTH 02060698), Chester Pass, Stirling Range, Bluff Knoll turnoff from Albany-Borden road, –, –;

*A. polyphylla* (8), *Briggs B.G. 9350a* (NSW), Near Lake King, –, AF148720;

*A. polyphylla* (9), *Briggs B.G. 9369* (PERTH 06156010), Eneabba Road 0.4 km from Brand Highway, Irwin District, –, –;

*A. polyphylla* (10), *Briggs B.G. 9524* (PERTH 06837581, 07312466), Brand Highway at entrance to Waslee Downs, 4 km SSE of Boothendara Creek, –, –;

*A. polyphylla* (11), *Briggs B.G. 9525* (PERTH 06829104, 07312474, 08012636), Brand Highway at entrance to Waslee Downs, 4 km SSE of Boothendara Creek, –, –;

*A. polyphylla* (12), *Briggs B.G. 9542* (PERTH 07313012), 10 km NE of Eneabba, –, –;

*A. polyphylla* (13), *Briggs B.G. 9543* (PERTH 07313071), c. 10 km NE of Eneabba, –, –;

*A. polyphylla* (14), *Briggs B.G. 9606* (PERTH 07312628), Brand Highway at entrance to Waslee Downs, 4 km SSE of Boothendara Creek, –, –;

*A. polyphylla* (15), *Briggs B.G. & Johnson L.A.S. BB 7813* (PERTH 02060655), Ca 8 km S of Lake Chidnup, 22.5 km N of Highway 1 on road to Lake King, –, –;

*A. polyphylla* (16), *Briggs B.G. & Johnson L.A.S. BB 7814* (PERTH 02060663), Ca 8 km S of Lake Chidnup, 22.5 km N of Highway 1 on road to Lake King, –, –;

*A. polyphylla* (17), *Briggs B.G. & Johnson L.A.S. BB 8510* (PERTH 01586130), 8 km SE of Fitzgerald Road on Aerodrome Road, ca 34 km NW of Ravensthorpe, –, –;

*A. polyphylla* (18), *Byrne G. 5419* (PERTH 08733872), Cairlocup Nature Reserve, gravel pit along Cowcher Road, –, –;

*A. polyphylla* (19), *Byrne G. 5420* (PERTH 08733880), Cairlocup Nature Reserve, gravel pit along Cowcher Road, –, –;

*A. polyphylla* (20), *Campbell R. 428* (PERTH 04891058), Corrigin, Fraser Job's location 25883, 2 km SW of Jubuk back road on rail reserve, –, –;

*A. polyphylla* (21), *Craig G.F. 5858* (PERTH 07540833), 18.5 - 19.5 km W of Lake King - Ravensthorpe Road on Aerodrome Road (= 0.3-1.4 km E of Long Creek Road intersection). Gravel pit No. 2, –, –;

*A. polyphylla* (22), *Cranfield R.J. & Spencer P.J. 8458* (PERTH 02849437), Hill River crossing Brand Highway, –, –;

*A. polyphylla* (23), *Davis R. 3096* (PERTH 04696379), Brookton - Corrigin Road (via Kweda), 10 km W of Kweda, –, –;

*A. polyphylla* (24), *Davis R. 3372* (PERTH 04907094), 900 m along road running E from intersection of Mount Adams and Tomkins Road, –, –;

*A. polyphylla* (25), *Fomichev C.I. et al. WA575* (MW 0940616), Brand Highway at entrance to Waslee Downs, 4 km SSE of Boothendara, *MT775998*, *MT775948*;

*A. polyphylla* (26), *Griffin E.A. 5250* (PERTH 03208311), Boothendarra Hill Reserve (29719) N of Badgingarra AMG 50JLM 613503 (Badgingarra 1:50,000 sheet), –, –;

*A. polyphylla* (27), *Griffin E.A. 7604* (PERTH 03510921), N of Mt Adams Rd, E of Yardanargo [Yardanogo] Nature Reserve, SE of Dongara, –, –;

*A. polyphylla* (28), *Hickman E. & Gilfillan S. EJH 1737* (PERTH 06921310), Long term monitoring plot 08VA, 0.5 km along firebreak running SW of Bluff Knoll Road, 0.3 km from Chester Pass Road in Stirling Range National Park, Gazetted Reserve 14732, –, –;

*A. polyphylla* (29), *Hnatiuk R.J. 770042,* (PERTH 02334933), SE of Lake Indoon, –, –;

*A. polyphylla* (30), *Hnatiuk R.J. 770331* (PERTH 02060647), Ca 16 km SE of Kulin, –, –;

*A. polyphylla* (31), *Hoggart M. & Adams E. EA 538* (PERTH 08708592), Northern firebreak of Lake Shaster Nature Reserve, –, –;

*A. polyphylla* (32), *Keighery G.J. 7637* (PERTH 03463621), Near Roe's Rock, Fitzgerald River National Park, –, –;

*A. polyphylla* (33), *Keighery G.J. 11404* (PERTH 01415654), Cheyne Beach, Bluff Creek Road, –, –;

*A. polyphylla* (34), *Keighery G.J. & Gibson N. 4861* (PERTH 06692338), On E side of Corackerup Road, 2 km N of Boxwood Hill Ongerup Road junction, Corackerup Nature Reserve, c. 35 km N of Wellstead. [Plot - ST19], –, –;

*A. polyphylla* (35), *Keighery G.J. & Gibson N. 4885* (PERTH 06849652), 160 m S of Aerodrome Road, 4.7 km SE of Fitzgerald Road, Aerodrome Road Nature Reserve, c. 43 km S of Lake King Townsite. [Plot - LK11], –, –;

*A. polyphylla* (36), *Maslin B.R. MDT 224* (PERTH 02060612), 11 miles E of Newdegate on the road to Lake King, –, –;

*A. polyphylla* (37), *Meissner R. KSV 98* (PERTH 05675383), Kent, –, –;

*A. polyphylla* (38), *Meissner R. LB 999* (PERTH 05777739), Reserve No:29020, Polygon No: 644602, Shire of Kent, –, –;

*A. polyphylla* (39), *Newbey K.R. 3723* (PERTH 02060620), 11 miles SE of Ongerup, –, –;

*A. polyphylla* (40), *Newbey K.R. 4077* (PERTH 02484730), 22.5 km SE of Borden, –, –;

*A. polyphylla* (41), *Newbey K.R. 4669* (PERTH 03714845), 4.5 km SE of Qualup Homestead (FRNP), –, –;

*A. polyphylla* (42), *Obbens F. 139/97* (PERTH 04964934), On vacant crown land or shire reserve, ca 2 km N along Greaves Hill road from junction with Kuch Road, near Pallinup River, NE of Albany, –, –;

*A. polyphylla* (43), *Sage L.W. et al. LWS 1272* (PERTH 05491967), Jingaring Nature Reserve, Jingaring road, c. 30 km ENE of Pingelly, –, –;

*A. polyphylla* (44), *Tindale M.D. 3762* (PERTH 02060728), 0.5 miles (1 km) E of Newdegate on the main road to Lake King, –, –;

*A. polyphylla* (45), *Turley C.D. 5 MONJ 596* (PERTH 05019982), Monjingup Lake near granite area, –, –;

*A. polyphylla* (46), *WA Herbarium WAH 334* (PERTH 08425698), Reserve 10147, Robinson Rd, 9.7 km west of Woodanilling, –, –;

*A. polyphylla* (47), *Wenham P. LG 2-11* (PERTH 05620619), Kent, –, –;

*A. polyphylla* (48), *Westcott V. S.M. 31 a* (PERTH 07824572), South Eneabba Nature Reserve, –, –;

*A. polyphylla* (49), *Westcott V. SM 44 a* (PERTH 07863047), South Eneabba Nature Reserve (NR 27886), 4.3 km S of Eneabba on Brand Highway, turn down firebreak heading W. After 300 m, site is located approx. 150 m S of the track, –, –;

*A. polyphylla* (50), *Wilson K.L. 2804* (PERTH 01415646), 19 km S of Lake King on Ravensthorpe, –, –;

*A. polyphylla* (51), *Wilson P.G. 6975* (PERTH 02060736), 8 km W of Lake King to Ravensthorpe road and 2 km S of Lake King township, –, –;

*A. polyphylla* (52), *Wilson P.G. 6982* (PERTH 02060604), 12 km W of Lake King-Ravensthorpe road and ca 20 km S of Lake King, –, –;

***Anarthria prolifera* R.Br.**

*A. prolifera* (1), *Fomichev C.I. & Macfarlane T.D. WA409* (MW), 2 km N from Bakers Junction NR, –, *MT775901*;

*A. prolifera* (2), *Fomichev C.I. & Macfarlane T.D. WA411a* (MW), Gull Rock NP, –, *MT775902*;

*A. prolifera* (3), *Fomichev C.I. et al. WA358B* (MW 0940624), Vasse Highway, *MT775955*, *MT775900*;

*A. prolifera* (4), *Meney K.A. s.n.* (NSW), Jarrahdale, –, GQ409009;

***Anarthria scabra* R.Br.**

*A. scabra* (1), *Briggs B.G. 9581* (NSW), Brockman Highway near Sues Rd, –, GQ408983;

*A. scabra* (2), *Fomichev C.I. & Macfarlane T.D. WA412* (MW), Gull Rock NP, –, *MT775903*;

*A. scabra* (3), *Fomichev C.I. & Macfarlane T.D. WA424* (MW), Albany, King River, –, *MT775904*;

*A. scabra* (4), *Sokoloff D.D. & Macfarlane T.D. WA25* (MW 0940635), Between Augusta and Albany, near Shannon Airstrip, *MT775956*, –;

***Hopkinsia adscendens* B.G.Briggs & L.A.S.Johnson**

*H. adscendens* (1), *Briggs B.G. 9342* (NSW), Oldfield River, –, AF148738;

*H. adscendens* (2), *Fomichev C.I. & Macfarlane T.D. WA389* (MW), c. 450 m SE of Marra Bridge, Pallinup River, *MT775950*, –;

*H. adscendens* (3), *Robinson C.J. & Woodall G.S. CJR1261* (PERTH 07386397), Slope into Oldfield River valley, east side of river, 500m south of highway, –, *MT775888*;

*H. adscendens* (4), *Sandiford E.M.* *2388* (PERTH 08915326), S bank of Pallinup River c. 400 m SE of Marra Bridge, South Coast Highway, –, *MT775889*;

***Hopkinsia anoectocolea* (F.Muell.) D.F.Cutler**

*H. anoectocolea* (1), *Fomichev C.I. et al. WA580* (MW 0940639), Arrowsmith River at Brand Highway, 10 m S from river, 50 m SW of Drummonds Bridge, *MT775951*, *MT775892*;

*H. anoectocolea* (2), *Hort F. & Hort J. FH4141* (PERTH 08979014), Private property access road, Mortlock River East Branch, 6 km at 62.57 deg. ENE from Meckering, –, *MT775891*;

*H. anoectocolea* (3), *Meney K.A. 920* (NSW 364831), Arrowsmith River, –, GQ409005;

*H. anoectocolea* (4), *Oversby B. BO47* (PERTH 05795826), Quellington Rd, 5 km SSW of Meckering, –, *MT775890*;

***Lyginia barbata* R.Br.**

*L. barbata* (1), *Briggs B.G. 9321* (NSW), N of Cataby, –, AF148748;

*L. barbata* (2), *Fomichev C.I. et al. WA316* (MW 0940579), Shannon Airstrip, *MT775953*, *MT775897*;

*L. barbata* (3), *Sokoloff D.D. & Macfarlane T.D. WA26* (MW 0940583), Between Augusta and Albany, South Western Highway and Beardmore Rd intersection, –, *MT775896*;

***Lyginia excelsa* B.G.Briggs & L.A.S.Johnson**

*L. excelsa* (1), *Briggs B.G. 9556* (PERTH 07312539), Brand Highway 105 km N of Gingin, –, *MT775893*;

*L. excelsa* (2), *Coultas D. DC-OPP05* (PERTH 08999112), Cadda Rd Reserve / Badgingarra NP, ca. 50 m S of Cadda Rd, ca. 10.7 km WSW from intersection of Cadda Rd and Brand Highway, –, *MT775894*;

*L. excelsa* (3), *Fomichev C.I. et al. WA574* (MW), Brand Highway, *MT775952*, *MT775895*;

***Lyginia imberbis* R.Br.**

*L. imberbis* (1), *Briggs B.G. 9477* (NSW), S of Arrowsmith River, –, GQ409006;

*L. imberbis* (2), *Sokoloff D.D. & Barrett M.D. WA109* (MW 0940592), NE of Jurien Bay, S of Jurien Rd and E of Black Arrow Rd, *MT775954*, *MT775899*;

*L. imberbis* (3), *Wheeler M. MW13* (PERTH 07729529), Namelcatchem NR, –, *MT775898*;

***Sporadanthus strictus* (R.Br.) B.G.Briggs & L.A.S.Johnson**

*Fomichev C.I. & Macfarlane T.D. WA383* (MW), South Western Highway and Beardmore Rd, *MT775949*, *MT775887*;
